# Danggui Buxue Decoction Attenuates *Staphylococcus aureus*-Induced Mastitis in Mice Associated with Gut Microbiota Remodeling, Blood–Milk Barrier Protection, and Inflammatory Suppression

**DOI:** 10.3390/vetsci13070613

**Published:** 2026-06-25

**Authors:** Qian Ma, Jiaqi Dong, Rong Yang, Yongli Hua, Fanlin Wu, Yanming Wei, Peng Ji

**Affiliations:** 1 Institute of Traditional Chinese Veterinary Medicine, College of Veterinary Medicine, Gansu Agricultural University, Lanzhou 730070, China; 2 Lanzhou Institute of Husbandry and Pharmaceutical Science, Chinese Academy of Agricultural Science, Lanzhou 730050, China

**Keywords:** Danggui Buxue decoction, mastitis, gut microbiota, NF-κB/NLRP3 pathway, MAPK pathway, blood–milk barrier

## Abstract

This study investigated the protective effects of DBD against *Staphylococcus aureus* (*S. aureus*)-induced mastitis in mice and explored whether these effects were associated with gut microbiota alterations, blood–milk barrier integrity, and inflammatory signaling. A lactating mouse model of mastitis was established, and the effects of DBD were evaluated using HPLC, histopathological analysis, ELISA, qRT-PCR, Western blotting, immunofluorescence, and 16S rRNA sequencing. The results showed that DBD significantly reduced bacterial loads in mammary tissues, decreased the expression of pro-inflammatory cytokines, including TNF-α, IL-1β, and IL-6, and alleviated inflammatory cell infiltration and tissue damage. Moreover, DBD upregulated the expression of tight junction proteins and improved the integrity of the blood–milk barrier. DBD treatment was also associated with alterations in gut microbiota composition, as reflected by changes in the relative abundance of several bacterial taxa. In addition, DBD inhibited the activation of the NF-κB/NLRP3 and MAPK inflammatory signaling pathways. Collectively, these findings indicate that DBD alleviates *S. aureus*-induced mastitis accompanied by alterations in gut microbiota composition, suppressing inflammatory responses, and repairing the blood–milk barrier, suggesting its potential as a therapeutic agent for mastitis.

## Simple Summary

Mastitis caused by *Staphylococcus aureus* is a common inflammatory disease that affects mammary gland health and dairy production. This study investigated whether DBD could protect against *S. aureus*-induced mastitis in lactating mice. The results showed that DBD reduced bacterial burden, inflammation, and tissue damage in the mammary gland. DBD also helped restore the blood–milk barrier, regulate gut microbiota composition, and inhibit key inflammatory signaling pathways. These findings suggest that DBD may be a promising natural therapeutic candidate for the prevention or treatment of mastitis.

## 1. Introduction

Mastitis ranks among the most impactful diseases in the dairy industry, as it drastically degrades milk production and impairs milk quality, causing considerable economic losses worldwide [1]. Among the various biological factors contributing to mastitis, *Staphylococcus aureus* (*S. aureus*) is recognized as the predominant infectious agent [2,3]. *S. aureus* drives disease development by identifying and attaching to Toll-like receptor 4 (TLR4) located on cellular membranes. This interaction subsequently activates significant downstream signaling cascades, specifically the nuclear factor-κB (NF-κB) pathway and mitogen-activated protein kinases (MAPKs) [4]. Furthermore, existing evidence indicates that the activation of the NLRP3 inflammasome can be induced by reactive oxygen species (ROS) and potassium ion (K^+^) outflow. The maturation of interleukin-1β (IL-1β) is, in turn, facilitated by this activation, a process that is governed by caspase-1-mediated processing [5]. It is well-documented that these coordinated signaling pathways act as the primary triggers for the secretion of pro-inflammatory cytokines—such as IL-6, IL-1β, and tumor necrosis factor-α (TNF-α). The combined effect of these cascades ultimately leads to the development or worsening of mastitis.

The involvement of gut microbiota in maintaining health and influencing disease progression has become an increasingly prominent focus of contemporary scientific inquiry [6]. A growing body of evidence suggests that gut microbial communities govern a broad spectrum of disease processes, covering diabetes, fatty liver disease, atherosclerosis, and cancer [7,8,9,10]. Recent studies have proposed that gut microbial dysregulation may exacerbate mammary inflammatory responses, possibly involving the “gut-mammary axis”. This insight highlights the potential relevance of microbiota–immune crosstalk in mammary inflammatory disease progression [11]. It is noteworthy that pomegranate flower polysaccharides (PFPS) have been reported to alleviate mastitis in association with improved microbial homeostasis. This was accompanied by a reduction in pathogenic bacteria and an increase in probiotic abundance. In that study, PFPS treatment was associated with reduced mastitis incidence and improved blood–milk barrier integrity [12]. Additionally, fecal microbiota transplantation (FMT) from mice treated with Astragalus polysaccharides (ASP) to recipient animals was reported to reduce the severity of *S. aureus*-associated mastitis. In the same study, the administration of oral ASP was associated with the enrichment of Lachnospiraceae_NK4A136 and suppression of Erysipelatoclostridium populations, indicating alterations in the gut microbial composition and diversity [13].

The blood–milk barrier represents a critical functional component within the mammary glands of mammals, playing an essential regulatory role in maintaining milk composition homeostasis and bodily defense. This barrier selectively regulates molecular exchange between blood and milk, thereby ensuring the nutritional integrity of milk while effectively preventing the invasion of pathogenic microorganisms and harmful substances [14]. Its structural foundation primarily consists of tight junctions (TJs)—highly dynamic, specialized junctional complexes between epithelial cells. The proper function of TJs depends on the expression and correct organization of core proteins, including occludin, claudin family members, and the cytoplasmic scaffolding protein ZO-1. Research findings have confirmed that the permeability of the blood–milk barrier is closely controlled by the structural integrity of TJs [15]. Furthermore, TJs seal the paracellular pathway, thereby blocking the translocation of pathogens into milk; thus, downregulation or structural abnormalities of TJ proteins can compromise barrier function. It has been established that pro-inflammatory agents like lipopolysaccharide (LPS) can markedly reduce the levels of TJ proteins, compromise the structural stability of the blood–milk barrier, raise the vulnerability of mammary tissue to pathogenic intrusion, and worsen mastitis severity [16]. Thus, preserving the structural completeness of TJs is essential for sustaining blood–milk barrier performance and alleviating inflammatory reactions in mastitis.

During the Jin-Yuan Dynasty, Li Dongyuan crafted Danggui Buxue Decoction. It consists of *Astragalus mongholicus* (*Fisch.*) *Bunge* and *Angelica sinensis* (*Oliv.*) *Diels* mixed at a 5:1 proportion; in Traditional Chinese Medicine (TCM), this formula is broadly acknowledged for its ability to concurrently tonify qi and nourish blood. Within the TCM theory, mastitis is primarily ascribed to liver qi stagnation and impaired flow of qi and blood [17]. *Astragalus mongholicus* is known to tonify qi and secure the exterior, whereas *Angelica sinensis* promotes blood circulation and unblocks collaterals. Their combination acts synergistically to alleviate local stagnation of qi and blood, consistent with the TCM pathogenesis principle that “obstruction leads to pain” [18]. Modern pharmacological studies have revealed multifaceted bioactivities of DBD, including enhanced haematopoiesis and cardiovascular protection [19,20]. Using HFLS-RA cell models in vitro and the collagen-induced arthritis (CIA) rat model, Xin et al. demonstrated that DBD significantly alleviated rheumatoid arthritis by inhibiting pro-inflammatory cytokine expression and modulating the Wnt/β-catenin pathway [8]. Empirical studies further reveal that DBD safeguards against acute lung injury triggered by LPS; according to Shi et al. [21] and X. Jiang et al. [8], it also affects Nrf2-driven oxidative stress responses and Nrf2-independent NLRP3/NF-κB signaling cascades. Independently, DBD can reduce inflammation in the lungs by blocking the TLR4-dependent p38 MAPK and NF-κB signaling cascades [22]. Some research also supports the efficacy of DBD in inhibiting NLRP3 activation, suggesting potential therapeutic benefits against peritonitis and gouty arthritis [23]. Conversely, Gong and co-workers documented that DBD was able to trigger the NF-κB pathway within macrophages, prompting the synthesis of pro-inflammatory mediators like IL-6, IL-8, TNF-α, and IL-1β—an effect that points to DBD’s role in immune regulation [24]. In rat models, DBD inhibits NLRP3 inflammasome activity and renal inflammation, indicating the potential applicability in mitigating tubulointerstitial fibrosis [25]. When it comes to intestinal wellness, DBD has been shown to ease symptoms of inflammatory bowel disease—it does so by regulating immune reactions and fostering recovery of the intestinal mucosal barrier [26]. Recent studies also highlight its efficacy in reducing stress-induced damage. For example, DBD reduced oxidative stress injury via activation of the Nrf-2 pathway, supporting its development as a therapeutic agent for liver conditions [27]. Moreover, it protects against chronic unpredictable mild stress-induced insulin resistance, partly by suppressing macrophage infiltration into pancreatic islets and stress-induced NLRP3 activation [28].

Given the above therapeutic benefits of DBD in disease management, it has emerged as a promising agent for addressing multiple inflammation-linked disorders. *S. aureus* acts as a major causative bacterium in chronic and subclinical mastitis cases. It is commonly employed as a model to study bacterial exposure in mammalian cell systems and further elicit the release of pro-inflammatory cytokines [27,29]. Nevertheless, no existing studies have explored the therapeutic potential of DBD against *S. aureus*-induced mastitis. To investigate this unexplored area, this study employed *S. aureus* as an inflammatory stimulant to systematically evaluate DBD’s protective effects on murine mastitis through integrated methodologies, including special staining techniques and molecular biological approaches, with particular emphasis on elucidating its underlying mechanisms.

## 2. Materials and Methods

### 2.1. Main Reagents

The crude herbs of Danggui (*Angelica sinensis*) and Huangqi (*Astragalus mongholicus*), both originating from Min County in China’s Gansu Province, were sourced from the Yellow River Herb Market, located in Lanzhou City. Calycosin-7-O-glucoside (batch no. Yz062824), Chlorogenic acid (batch no. Yz1008201), Ononin (batch no. Yz100623), Ferulic acid (batch no. Yz011422), and Formononetin (batch no. Yz0104201) were supplied by Nanjing Yuanzhi Biotechnology Co., Ltd (Nanjing China). The bacterial strain Staphylococcus aureus (ATCC 29213) was procured from the American Type Culture Collection (ATCC, Manassas, VA, USA). Dexamethasone was a generous gift from the First Hospital of Lanzhou University, China. Several reagents, including RIPA buffer (R0010), a BCA protein assay kit (PCO020), and pre-stained protein markers (G2087), were acquired from Solarbio Science & Technology Co., Ltd. Beijing, China, for the present study. Additionally, SDS-PAGE loading buffer (5×, P1041) was provided by Solarbio Technology Co., Ltd. (Beijing, China).

The following primary antibodies were used: NLRP3 (#15101; Cell Signaling Technology, Inc. Danvers, MA, USA), ASC (bs-6741R; Bioss, Beijing, China), Caspase-1 (22915-1-AP; Proteintech, Wuhan, China), p-IκBα (82349-1-RR; Proteintech), p-p65 (10745-1-AP; Proteintech), iNOS (22226-1-AP; Proteintech), β-actin (2536-1-AP; Proteintech), p65 (T55034; ABmart, Shanghai, China), IκBα (T55026; ABmart), ERK (T40071; ABmart), p-ERK (PC3292; ABmart), JNK (T40073; ABmart), p38 (T55600; ABmart), GAPDH (P30008S; ABmart), p-JNK (WL01813; Wanleibio, Shenyang, China), p-p38 (WLP1576; Wanleibio), MPO (WL02355; Wanleibio), and Cox-2 (WL01750; Wanleibio). HRP-conjugated goat anti-rabbit IgG secondary antibody (Proteintech Group, SA00001-2) was also used.

Solarbio Technology Co., Ltd. (Beijing, China) supplied the BCA protein assay kit (PCO020). Furthermore, the following secondary antibodies were utilized: HRP-conjugated AffiniPure goat anti-rabbit/mouse IgG (H+L) (SA00001-2; Proteintech) and CoraLite^®^ 488-conjugated goat anti-rabbit/mouse IgG (H+L) (SA000013-2, SA000013-1; Proteintech).

### 2.2. Major Instruments

This study employed the following instruments: a DZ5-WS multi-tube automatic balance centrifuge (Changsha Xiangyi Centrifuge Instrument Co., Ltd., Changsha, China); a SpectraMax 340PC384 microplate reader (Molecular Devices, San Jose, CA, USA); an RM2245 rotary microtome (Leica, Biosystems, Wetzlar, Germany); an Olympus NE910 microscopic imaging system (Olympus, Corporation, Tokyo, Japan); an Amersham Image Quant 800 chemiluminescence imaging system (Cytiva, Marlborough, MA, USA); and a high-resolution mass spectrometer of the Q Exactive HFX model (Thermo Fisher Scientific Inc., Waltham, MA, USA).

### 2.3. Preparation of DBD

DBD was prepared by combining *Astragalus mongholicus* and *Angelica sinensis* at a mass ratio of 5:1, according to the standard decoction protocol prescribed in the Chinese Pharmacopeia. The plant names were verified using http://www.worldfloraonline.org. The blended herbal materials were soaked in 10 volumes of distilled water (*v*/*w*) for 30 min and then extracted under reflux for 1 h. After filtration, the residual herbal materials were re-extracted under reflux with 8 volumes of distilled water (*v*/*w*) for 40 min, followed by a second filtration. The two filtrates were combined and centrifuged at 3000 rpm for 15 min. The supernatant was concentrated using a vacuum rotary evaporator at 60 °C to a final crude drug concentration of 1.00 g/mL and stored at 4 °C until further use.

### 2.4. Establishment of Animal Models

KM mice of both sexes (72 females and 36 males), aged 6–8 weeks, were sourced from the Experimental Animal Center of Lanzhou Veterinary Research Institute, Chinese Academy of Agricultural Sciences (Animal Use License: SCXK (Gan) 2022–0002). Following an acclimation period of 2–3 days, to enable mating, mice of both sexes were co-housed with two females paired with one male. Experiments were conducted using lactating females at 5–7 days post-delivery. Ultimately, 60 healthy lactating female mice that had successfully delivered pups and showed normal lactation status were included in the formal experiment and randomly assigned to six groups, with 10 mice per group. Unless otherwise stated, each mouse was considered one biological replicate, and the exact sample sizes for each analysis are indicated in the corresponding figure legends. All mice were kept under standardized laboratory conditions, which included an ambient temperature of relative humidity ranging from 40% to 80% and a 12 h light/12 h dark cycle. Food and water were provided ad libitum throughout the study.

The experimental design comprised six groups. These groups included a PBS-administered blank control, an *S. aureus* challenge group (1 × 10^7^ CFU/mouse), a low-dose Danggui Buxue Decoction (DBD) + *S. aureus* group, a medium-dose DBD + *S. aureus* group, a high-dose DBD + *S. aureus* group, and a dexamethasone (5.0 mg/kg body weight) + *S. aureus* group. Dexamethasone was used as a pharmacological positive anti-inflammatory control to verify the responsiveness of the mastitis model to an established anti-inflammatory intervention rather than for a direct efficacy comparison with DBD. Mice in the PBS and *S. aureus* groups received a daily oral gavage of physiological saline. Mice assigned to the DBD treatment groups were administered DBD by oral gavage once daily at doses of 3, 6, and 9 g/kg for the low-, medium-, and high-dose groups, respectively, for 6 consecutive days before the *S. aureus* challenge; these doses were expressed as crude herb equivalents. The dexamethasone + *S. aureus* group received an intraperitoneal injection of dexamethasone at 5.0 mg/kg body weight 1 h before modeling, and daily oral saline for 6 consecutive days. All mice, with the exception of those in the PBS control group, were administered a *S. aureus* bacterial suspension through the mammary duct on the seventh day of the experiment, establishing an infection model based on previously reported methods [30]. Twenty-four hours post-infection, the animals were humanely euthanized via cervical dislocation. Mammary gland samples were then harvested for subsequent analysis: one portion was preserved in 10% formalin for histological examination, while the remainder was snap-frozen at −80 °C for molecular studies. Concurrently, fecal samples were collected and stored at −80 °C to facilitate gut microbiota profiling. All assays were performed using samples from the same cohort of experimental animals, and the numbers of biological replicates used for each assay are indicated in the corresponding figure legends. The DBD dose selection was based on body surface area conversion from the clinical dose used in dairy cows. The clinical daily dose of DBD for lactating dairy cows, with an average body weight of 600 kg, is 144 g/day, expressed as a crude herb weight, corresponding to 0.24 g/kg. Using Km values of 111.8 for cows and 3 for mice, the mouse equivalent dose was calculated to be 8.94 g/kg. Therefore, 9 g/kg was selected as the high dose, while 3 g/kg and 6 g/kg were set as the low and medium doses, respectively. As the high dose approximates the mouse equivalent dose converted from the clinical dose in dairy cows, it was considered physiologically and clinically relevant.

### 2.5. High-Performance Liquid Chromatography (HPLC) Analysis

An Agilent HPLC system fitted with a Zorbax SB C18 column (250 mm × 4.6 mm, 5 μm) was used for the compositional analysis of DBD. Gradient elution was performed using water containing 0.1% phosphoric acid (A) and acetonitrile (C) as the mobile phase. The elution program was as follows: 25–32% C, 0–9 min; 32–40% C, 9–14 min; 40–45%, 14–16 min; and 45–50% C, 6–19 min. The flow rate was maintained at 1.0 mL/min, and detection was performed at 280 nm. The column temperature was maintained at 35 °C, and the injection volume was 20 μL.

For quantitative analysis, external standard calibration curves were established for calycosin-7-O-glucoside, chlorogenic acid, ononin, ferulic acid, and formononetin under the same chromatographic conditions. In addition, HPLC fingerprint analysis was performed using 11 batches of DBD samples, and a similarity evaluation was conducted using the Similarity Evaluation System for Chromatographic Fingerprint of Traditional Chinese Medicine, version 2012.130723. Detailed calibration equations, linear ranges, fingerprint profiles, similarity evaluation, and quantitative results are provided in the Supplementary Materials.

### 2.6. Bacterial Load in the Mammary Gland

To assess bacterial colonization in the mammary gland, 1 mL of PBS was combined with 0.1 g of tissue samples, and then homogenized using a homogenizer. The resulting homogenate was then subjected to analysis via a 10-fold serial dilution technique. Aliquots of 100 μL from the diluted samples were plated onto nutrient agar, with triplicate plates prepared for each experimental group. *S. aureus* colonies were enumerated following a 24 h incubation at 37 °C, with the results presented as colony-forming units (CFU).

### 2.7. Histopathological Analysis

Mammary tissue samples were first dissected into smaller fragments and immersion-fixed in 10% formalin. Subsequently, the tissue samples underwent a sequence of processing steps: sequential dehydration in a graded ethanol series, clearing with xylene, and final embedding in paraffin wax. Subsequently, thin slices were cut from the resultant blocks and subjected to standard hematoxylin and eosin (H&E) staining. Observation of histopathological alterations and image acquisition were carried out using a conventional light microscope.

### 2.8. Immunohistochemistry (IHC) Analysis

Following a standard deparaffinization and rehydration procedure, sections of paraffin-embedded mouse mammary tissue were prepared for immunohistochemistry. Endogenous peroxidase activity was quenched by treatment with a peroxidase blocking reagent. Antigen retrieval was conducted using citrate buffer, and non-specific binding sites were pre-blocked with 5% bovine serum albumin (BSA) solution. The sections were exposed to a primary antibody targeting MPO (1:2000 dilution) and incubated overnight at 4 °C. After thorough washing, the tissue was treated with a goat anti-rabbit IgG secondary antibody for 30 min at 37 °C. Signal detection was performed using diaminobenzidine (DAB) as the chromogenic substrate, followed by counterstaining with hematoxylin. After completing staining, the tissue slices were dehydrated and cleared, and then affixed to coverslips with neutral gum to support subsequent microscopic observation and image capture.

### 2.9. Determination of Inflammatory Cytokines by ELISA

Mammary tissue samples were processed with PBS at a 1:9 (*w*/*v*) ratio utilizing a mechanical homogenizer. Following centrifugation of the homogenate at 3000 rpm for 20 min, the supernatant was collected for subsequent assays. Total protein content was quantified via the bicinchoninic acid (BCA) method. Concentrations of MPO, TNF-α, IL-1β, and IL-6 were then determined in the mammary tissue extracts using ELISA kits, adhering closely to the operational protocols advised by the manufacturer.

### 2.10. Gene Expression Profiles of Inflammatory Cytokines Were Evaluated by Quantitative Real-Time PCR (qRT-PCR)

Total RNA was isolated from mammary tissue samples with TRIzol reagent. Subsequent steps involved genomic DNA elimination and first-strand cDNA synthesis, which were carried out using the EasyScript^®^ One-Step gDNA Removal and cDNA Synthesis SuperMix (AE341-02, TransGen Biotech, Co., Ltd. Beijing, China). Amplification reactions were performed with PerfectStart^®^ Green qPCR SuperMix (AQ602-02, TransGen Biotech, Co., Ltd., Beijing, China) on a thermal cycler programmed as follows: The reaction protocol comprised an initial denaturation at 94 °C for 30 s, followed by 40–45 cycles of denaturation at 94 °C for 5 s, and annealing/extension at 60 °C for 30 s, concluding with a final melt curve analysis. All primer sequences used are listed in Table 1 and were designed as well as synthesized by Sangon Biotech (Shanghai, China).

### 2.11. Detection and Analysis of Gut Microbiota

Total genomic DNA from the microbial population was extracted using the E.Z.N.A.^®^ Soil DNA Kit (Omega Bio-tek, Norcross, GA, USA) according to the manufacturer’s recommended methods, and its quality was assessed. Thereafter, polymerase chain reaction (PCR) was conducted to amplify the V3–V4 hypervariable regions of the bacterial 16S rRNA gene, utilizing the purified DNA as a template, with barcoded primers 338F (5′-ACTCCTACGGGAGGCAGCAG-3′) and 806R (5′-GGACTACHVGGGTWTCTAAT-3′). After purification and quantification, the amplicons were pooled into equimolar ratios for library construction. Sequencing was performed using the Illumina Miseq PE300 or NovaSeq PE250 systems (Illumina, Inc., San Diego, CA, USA), with 10 replicates included per experimental group. The Majorbio Cloud Platform was employed for the bioinformatic analysis of the 16S rRNA sequencing data. For the assessment of alpha diversity, essential metrics included the Chao1 and Shannon indices, which were computed using Mothur software, and group differences were assessed via the Wilcoxon rank-sum test. Beta diversity was evaluated through principal coordinates analysis (PCoA) based on Bray–Curtis distances, and permutational multivariate analysis of variance (PERMANOVA) was conducted to examine the structural variations in microbial community composition among groups. Additionally, LEfSe was employed to detect bacterial taxa with differential abundance at the phylum to genus level, applying a significance threshold of LDA > 4 and a *p* < 0.05.

### 2.12. Transmission Electron Microscopy Analysis

Tissue specimens preserved at 4 °C were post-fixed with 1% osmium tetroxide. Dehydration was carried out through a graded acetone series (30%, 50%, 70%, 80%, 90%, 95%, and 100%) and three repetitions using pure acetone. The samples were subsequently infiltrated with progressively increasing concentrations of 812 epoxy resin diluted in acetone, using volume ratios of 3:1, 1:1, and 1:3, with each step maintained for 30–60 min. Following infiltration, the specimens were embedded in pure resin. Ultrathin sections, approximately 60–90 nm thick, were generated using an ultramicrotome, collected from the water surface in the knife boat, and mounted onto copper grids. The grids were then stained with uranyl acetate for 15–20 min and subsequently with lead citrate for 1–2 min at ambient temperature. Imaging was performed using a JEM-1400PLUS transmission electron microscope (JEOL Ltd., Akishima, Tokyo, Japan).

### 2.13. Immunofluorescence Assay

For immunofluorescence staining, tissue sections were initially deparaffinized, followed by antigen retrieval in EDTA buffer (pH 9.0). After rinsing with PBS, the sections were blocked with 5% bovine serum albumin (BSA) for 30 min. The sections were then incubated with the primary antibody overnight at 4 °C. Following another PBS wash, the sections were incubated with a fluorescence-labeled secondary antibody and kept in the dark for 30 min. Lastly, after a final PBS rinse, the nuclei were stained with DAPI, and the sections were mounted onto coverslips for microscopic visualization. Images from all groups were acquired under identical microscope settings. The mean fluorescence intensity was quantified using Images analysis software in three randomly selected fields for each group.

### 2.14. Western Blot Analysis

Total protein was extracted from mammary gland tissue using a high-efficiency RIPA lysis buffer. Following separation by SDS-PAGE, the extracted proteins were subsequently transferred onto PVDF membranes (Millipore, MA, USA). Membranes were blocked with 5% bovine serum albumin (BSA) for 2 h, followed by overnight incubation at 4 °C with specific primary antibodies. The primary antibodies used targeted IkBα, p-IkBα, p65, p-p65, NLRP3, Caspase-1, ASC, ERK, p-ERK, JNK, p-JNK, p38, p-p38, Claudin, Occludin, ZO-1, and β-actin. After washing, the membranes were probed with appropriate HRP-conjugated secondary antibodies for 1 h. Protein bands were visualized using an enhanced chemiluminescence (ECL) detection system, and band intensities were quantified using ImageJ software (version 1.54j). Non-phosphorylated proteins were normalized to β-actin, whereas phosphorylated proteins were normalized to their corresponding total protein levels.

### 2.15. Statistical Analysis

The results are presented as mean ± SEM. Statistical analyses and graphing were performed using GraphPad Prism software (version 9.0). Before statistical analysis, data normality was assessed using the Shapiro–Wilk test, and homogeneity of variance was evaluated using the Brown–Forsythe test. For normally distributed continuous data with homogeneous variance, multiple group comparisons were performed using one-way analysis of variance (ANOVA), followed by Tukey’s post hoc test.

For data that did not meet these assumptions, appropriate non-parametric tests were used. For microbiota data, statistical methods were selected according to the data type and distribution. Beta diversity was assessed by principal coordinate analysis (PCoA), and differences in microbial community structure were evaluated using analysis of similarities (ANOSIM). Differential microbial taxa were identified using the linear discriminant analysis effect size (LEfSe). A *p* < 0.05 was considered statistically significant. Pearson correlation analysis was performed to assess associations between microbial taxa and protein levels.

## 3. Results

### 3.1. Analysis of Chemical Constituents in DBD

**Figure 1:**
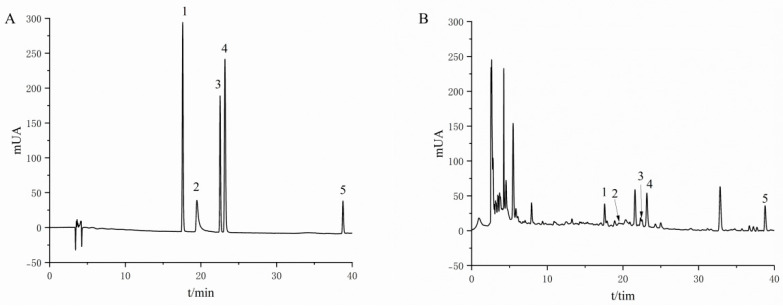
Chemical composition analysis of Danggui Buxue Decoction (DBD). 1: Calycosin-7-O-glucoside; 2: Chlorogenic acid; 3: Ononin; 4: Ferulic acid; 5: Formononetin. (**A**) Mixed reference standards; (**B**) HPLC chromatogram of the DBD test sample.

High-performance liquid chromatography (HPLC) was employed for the identification and quantification of two active compounds. Figure 1 illustrates the chromatograms obtained from the standard reference and the DBD sample. The analysis exhibited effective resolution of the target peaks with a consistent baseline, and the retention times in the sample closely matched those of the reference standards. The retention times of calycosin-7-O-glucoside, chlorogenic acid, ononin, ferulic acid, and formononetin in the mixed reference standards were 17.591 min, 19.470 min, 22.538 min, 23.178 min, and 38.780 min, respectively, while the corresponding values in the test sample were 17.596 min, 19.477 min, 22.323 min, 23.158 min, and 38.786 min, respectively.

Quantitative analysis was further conducted using external standard calibration curves. The five marker compounds showed good linearity, with correlation coefficients ranging from 0.9990 to 1.0000. Their concentrations in the prepared DBD sample solution were 0.0080 ± 0.0014, 0.0058 ± 0.0023, 0.0062 ± 0.0012, 0.0099 ± 0.0016, and 0.0957 ± 0.0287 mg/mL, respectively. HPLC fingerprint analysis of 11 batches of DBD samples showed similarity values greater than 0.98, indicating good batch-to-batch consistency. Detailed calibration equations, linear ranges, fingerprint profiles, similarity evaluation, and quantitative results are provided in the Supplementary Materials.

### 3.2. DBD Ameliorates S. aureus-Induced Murine Mastitis

**Figure 2:**
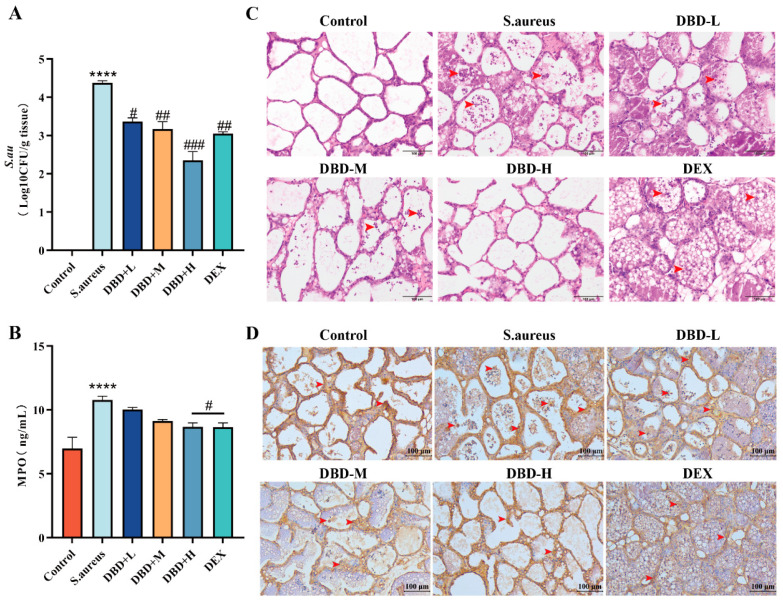
DBD attenuates *S. aureus*-induced mastitis in mice. (**A**) Bacterial load in mammary tissues. (**B**) MPO activity in mammary tissues. (**C**) Mammary tissue injury detected by H&E staining (scale bar = 100 μm; 200 × magnification). (**D**) Distribution of MPO in mammary tissues observed by immunohistochemical staining (scale bar = 100 μm; 200 × magnification). (n = 3 group). **** *p* < 0.0001 vs. control group; ^#^ *p* < 0.05, ^##^ *p* < 0.01, ^###^ *p* < 0.001 vs. *S. aureus* group.

To evaluate bacterial colonization, mammary gland samples were harvested at 24 h post-infection. No bacterial colonies were observed in the control animals. All DBD-treated groups exhibited a markedly lower bacterial burden in mammary tissue compared to the *S. aureus*-infected group, with high statistical significance (*p* < 0.01; Figure 2A).

H&E staining revealed intact mammary acinar structures and no significant pathological changes in the lumina of mice in the control group. The *S. aureus*-infected group exhibited marked inflammatory cell infiltration, with extensive diffuse infiltration of neutrophils and macrophages in the acinar lumina, mammary ducts, perivascular areas, and connective tissues, accompanied by structural damage. In contrast, DBD pretreatment markedly alleviated these pathological manifestations, reduced inflammatory cell infiltration, and improved the structural integrity (Figure 2C).

ELISA and immunohistochemical analyses results showed no neutrophil infiltration or significant inflammatory response in the control group. Compared with the control, the *S. aureus* group displayed substantial brown-yellow granular deposits and highly significantly elevated MPO activity (*p* < 0.01). After DBD intervention, stained areas were significantly reduced, and MPO activity was significantly decreased (*p* < 0.05), suggesting that DBD can alleviate inflammatory damage in mammary tissue by inhibiting MPO activity and neutrophil infiltration (Figure 2B,D).

**Figure 3:**
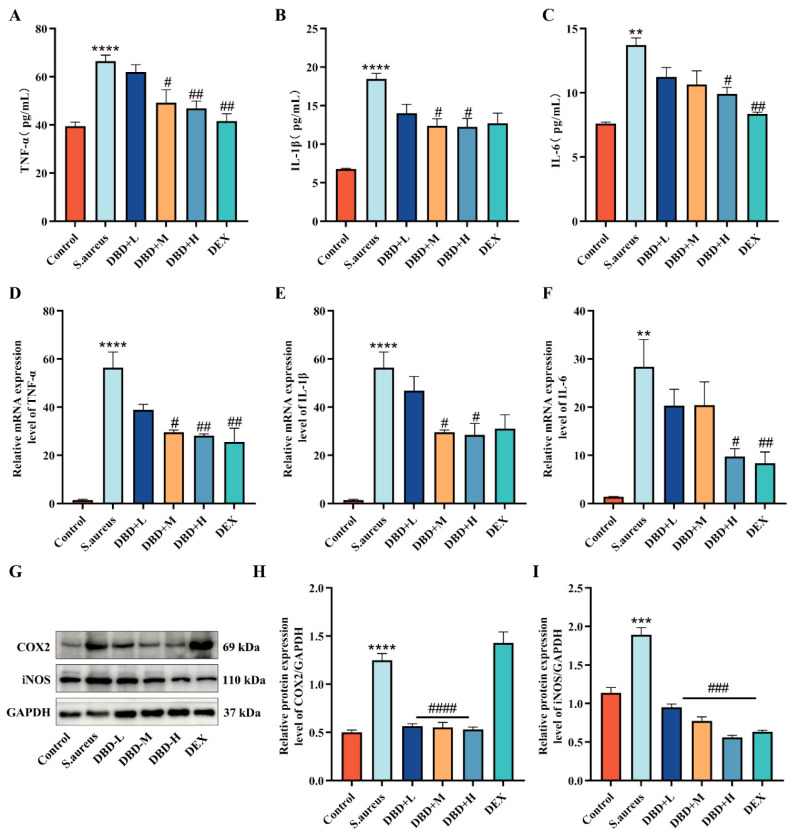
DBD alleviates *S. aureus*-induced mastitis in mice. (**A**–**C**) Protein expression levels of TNF-α, IL-1β, and IL-6 detected by ELISA. (**D**–**F**) mRNA levels of pro-inflammatory factors *TNF-α*, *IL-1β*, and *IL-6* measured using qRT-PCR. (**G**–**I**) Protein levels of COX-2 and iNOS detected by Western blot, with relative intensities of COX-2 and iNOS quantified. ((**A**–**C**): n = 5/group; (**D**–**I**): n = 3/group) ** *p* < 0.01, **** *p* < 0.0001 vs. control group; ^#^ *p* < 0.05, ^##^ *p* < 0.01, ^###^ *p* < 0.001, ^####^ *p* < 0.0001 vs. *S. aureus* group.

According to ELISA, mammary tissue concentrations of TNF-α, IL-1β, and IL-6 were markedly elevated following *S. aureus* infection (*p* < 0.01). Administration of DBD resulted in a dose-dependent suppression of these inflammatory mediators (*p* < 0.01), with the most substantial reduction observed at the highest dose (Figure 3A–C). Correspondingly, qRT-PCR analysis confirmed a pronounced upregulation of mRNA expression for these cytokines in the infected group (*p* < 0.01), which was also attenuated by DBD in a dose-related manner (*p* < 0.01). The high-dose regimen produced the strongest transcriptional inhibition, exceeding the effects of medium and low doses and aligning with the protein expression data (Figure 3D–F). Western blot further showed that infection significantly increased COX-2 and iNOS protein levels (*p* < 0.01), an effect that was progressively reversed by DBD pretreatment (*p* < 0.01). Again, the high-dose group displayed the most potent inhibitory action on both proteins (Figure 3G,H).

### 3.3. Effect of DBD on Gut Microbiota Diversity in Mice with S. aureus Mastitis

**Figure 4:**
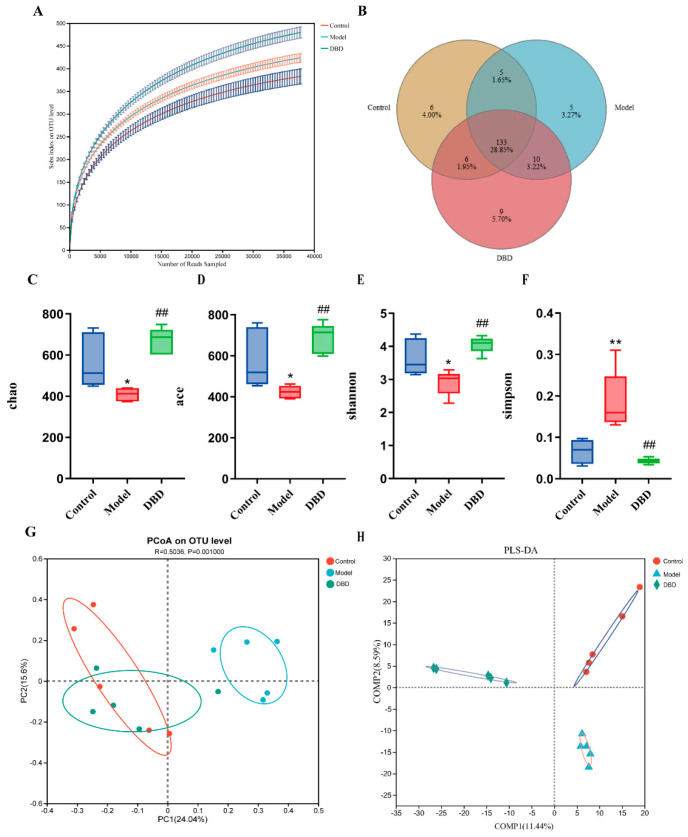
Effects of DBD treatment on gut microbiota diversity. (**A**) Species dilution curves. (**B**) Venn diagram of gut microbiota. (**C**–**F**) Alpha diversity indices, including the Chao1, ACE, Shannon, and Simpson indices. (**G**) Principal coordinates analysis (PCoA) based on OTUs. (**H**) Partial least squares-discriminant analysis (PLS-DA). (n = 5/group) * *p* < 0.05, ** *p* < 0.01 vs. control group; ^##^ *p* < 0.01 vs. *S. aureus* group.

High-throughput 16S rRNA sequencing was performed to characterize the microbial communities in cecal content samples from the control, model, and high-dose DBD groups. Rarefaction curves approached a plateau in all groups (Control, *S. aureus*, and DBD; DBD refers to the high-dose DBD group, DBD-H, in the microbiota analysis), indicating that the sequencing depth adequately captured the majority of microbial species present and supporting the reliability of subsequent compositional analyses (Figure 4A). Venn diagram analysis identified 133 operational taxonomic units (OTUs) that were common to all three groups, representing 28.85% of the total observed OTUs. The model group contained five unique OTUs, whereas the DBD group exhibited nine unique OTUs, suggesting group-specific differences in microbial composition (Figure 4B). Alpha diversity analysis showed that the Chao1, ACE, Shannon, and Simpson indices were decreased in the *S. aureus* group compared with the control group. (*p* < 0.05). Following DBD intervention, these indices were significantly elevated (*p* < 0.01), indicating that DBD treatment was associated with higher microbial richness and diversity that had been diminished by infection (Figure 4C,F). Principal coordinates analysis demonstrated distinct clustering of the model group separately from both control and DBD groups, with the DBD group showing partial overlap with the controls, indicating that DBD administration was associated with alterations in the overall gut microbial community structure. (Figure 4G). PLS-DA further supported the separation of microbial community profiles among the three groups (Figure 4H).

**Figure 5:**
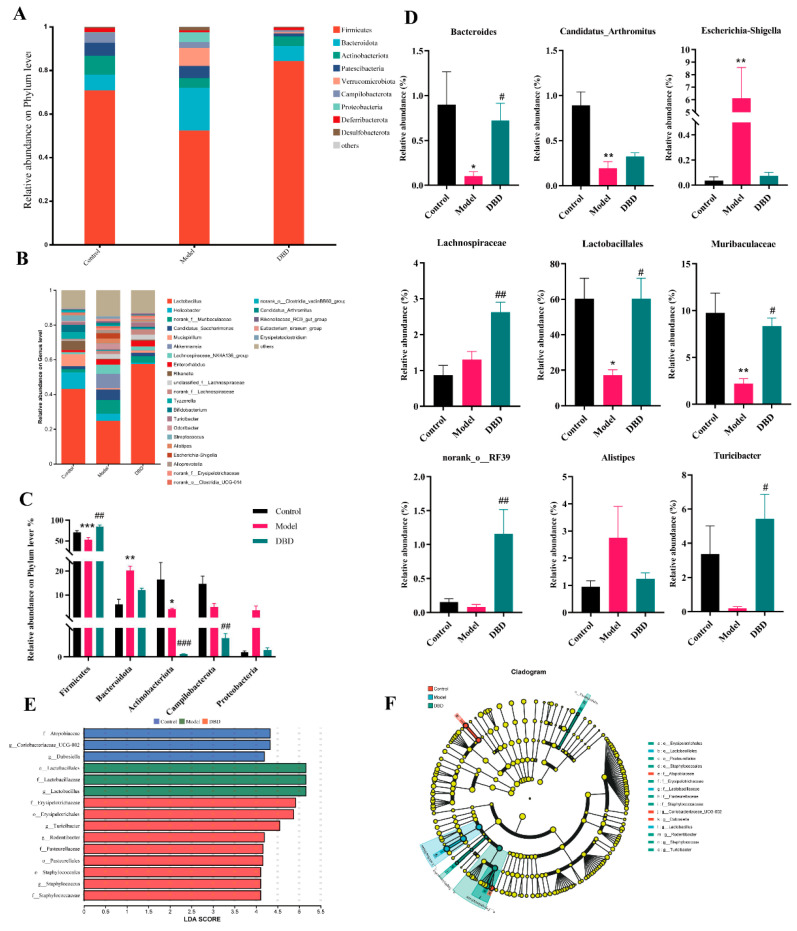
Effects of DBD treatment on gut microbiota composition and differentially abundant taxa. (**A**,**B**) Relative abundance histograms at the phylum and genus levels. (**C**,**D**) Differential analysis of gut microbiota at the phylum and genus levels. (**E**,**F**) LDA score plot and cladogram from LEfSe analysis showing microbial taxa with significant differences among groups. An LDA score > 4 was considered statistically significant. (n = 5/group) * *p* < 0.05, ** *p* < 0.01, *** *p* < 0.001 vs. control group; ^#^ *p* < 0.05, ^##^ *p* < 0.01, ^###^ *p* < 0.001 vs. *S. aureus* group.

Microbiota composition analysis at the phylum level revealed Firmicutes as the predominant phylum in both control and DBD groups, whereas the *S. aureus* group exhibited a co-dominance of Firmicutes and Bacteroidota (Figure 5A,C). Statistical comparisons showed that the model group had substantially reduced relative abundances of Firmicutes and Actinobacteria (*p* < 0.05), alongside a marked increase in Bacteroidota (*p* < 0.01) relative to the controls. Compared with the *S. aureus* group, DBD administration was associated with increased relative abundances of Firmicutes and Actinobacteria and a decreased relative abundance of Bacteroidota. (Figure 5C). At lower taxonomic levels, *Lactobacillales* predominated in the control and DBD groups, while the model group was characterized by the prevalence of *Lactobacillales*, g__norank_f__*Muribaculaceae*, and *Akkermansia* (Figure 5B). *S. aureus* infection significantly decreased the relative Bacteroides, *Candidatus*_*Arthromitus*, *Lactobacillales*, and *Muribaculaceae* (*p* < 0.05), while promoting a pronounced expansion of Escherichia-Shigella (*p* < 0.01). Compared with the model group, DBD administration was associated with shifts in these taxa, including increased relative abundances of Bacteroides, Candidatus_Arthromitus, Lactobacillales, and Muribaculaceae and a decreased relative abundance of Escherichia-Shigella (Figure 5D).

LEfSe analysis was performed to identify discriminative microbial taxa across the experimental groups. The control group was characterized by f__Atopobiaceae, g__Coriobacteriaceae_UCG-002, and g__*Dubosiella*, which belong to the phyla Actinobacteria and Firmicutes. The *S. aureus* group was characterized by several taxa within Firmicutes taxa o__Lactobacillales, f__Lactobacillaceae, and g__*Lactobacillus*, which were identified as discriminative taxa among the three groups. In the DBD group, the main discriminant taxa included f__Erysipelotrichaceae, o__Erysipelotrichales, g__*Turicibacter*, g__*Rodentibacter*, f__Pasteurellaceae, o__Pasteurellales, o__Staphylococcales, g__*Staphylococcus*, and f__Staphylococcaceae (Figure 5E,F).

### 3.4. Correlation Analysis Between Mammary Inflammatory Factors and Differential Gut Microbiota

**Figure 6:**
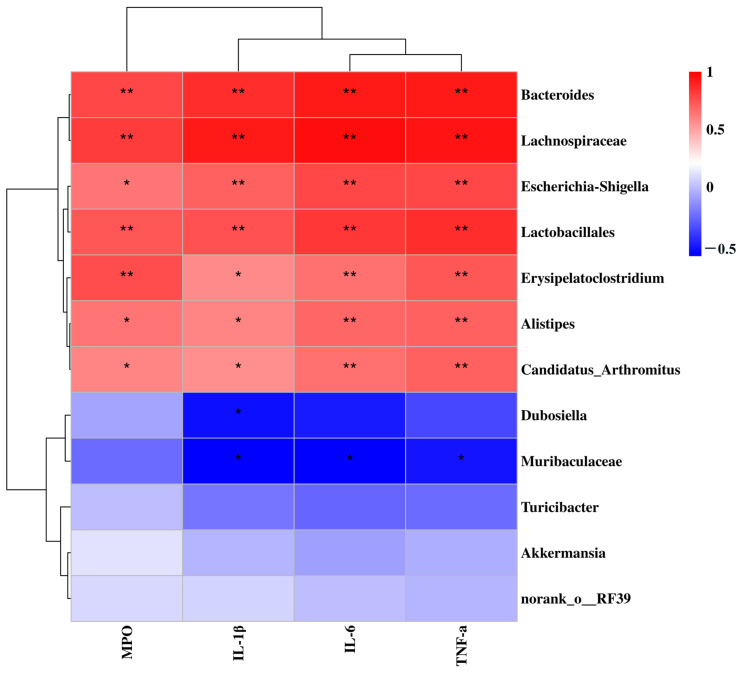
Pearson correlation analysis between differentially abundant intestinal microbial taxa and inflammatory mediators in mammary tissue. * *p* < 0.05, ** *p* < 0.01 based on unadjusted *p*-values.

To explore the possible association between intestinal microbiota and mammary gland inflammatory responses, Pearson correlation analysis was performed to assess the relationships between major inflammatory mediators in mammary tissue and differentially abundant microbial taxa. Based on unadjusted *p*-values, several nominally significant correlations were observed. The analysis indicated positive correlations between the MPO, IL-1β, IL-6, and TNF-α levels and the abundances of *Lachnospiraceae*, Bacteroides, Escherichia-Shigella, *Lactobacillales*, *Erysipelatoclostridium*, *Alistipes*, and Candidatus *Arthromitus*. In contrast, IL-1β exhibited a negative correlation with *Dubosiella* and *Muribaculaceae*, and similar inverse relationships were observed for IL-6 and TNF-α with *Muribaculaceae* (Figure 6). These findings suggest that alterations in gut microbial composition may be associated with *S. aureus*-induced mammary inflammatory responses, and that the protective effects of DBD against mastitis may be related, at least in part, to changes in inflammation-associated intestinal microbial taxa.

### 3.5. DBD Alleviates S. aureus-Induced Tight Junction Damage in Mouse Mammary Tissue

**Figure 7:**
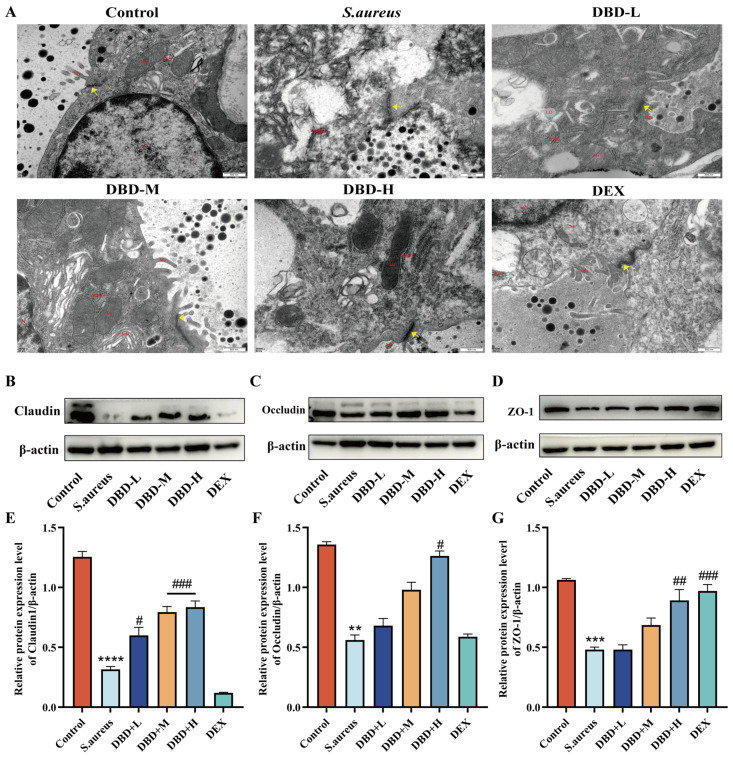
Effect of DBD on *S. aureus*-induced tight junction injury in mouse mammary tissue. (**A**) Representative transmission electron microscopy (TEM) images of tight junction structures in mammary tissue. (**B**–**D**) Expression of tight junction proteins (claudin-1, occludin, and ZO-1) in mammary tissue detected by Western blot. (**E**–**G**) Relative intensity of claudin-1, occludin, and ZO-1. ** *p* < 0.01, *** *p* < 0.001, **** *p* < 0.0001 vs. control group; (n = 3/group) ^#^ *p* < 0.05, ^##^ *p* < 0.01, ^###^ *p* < 0.001, vs. *S. aureus* group.

**Figure 8:**
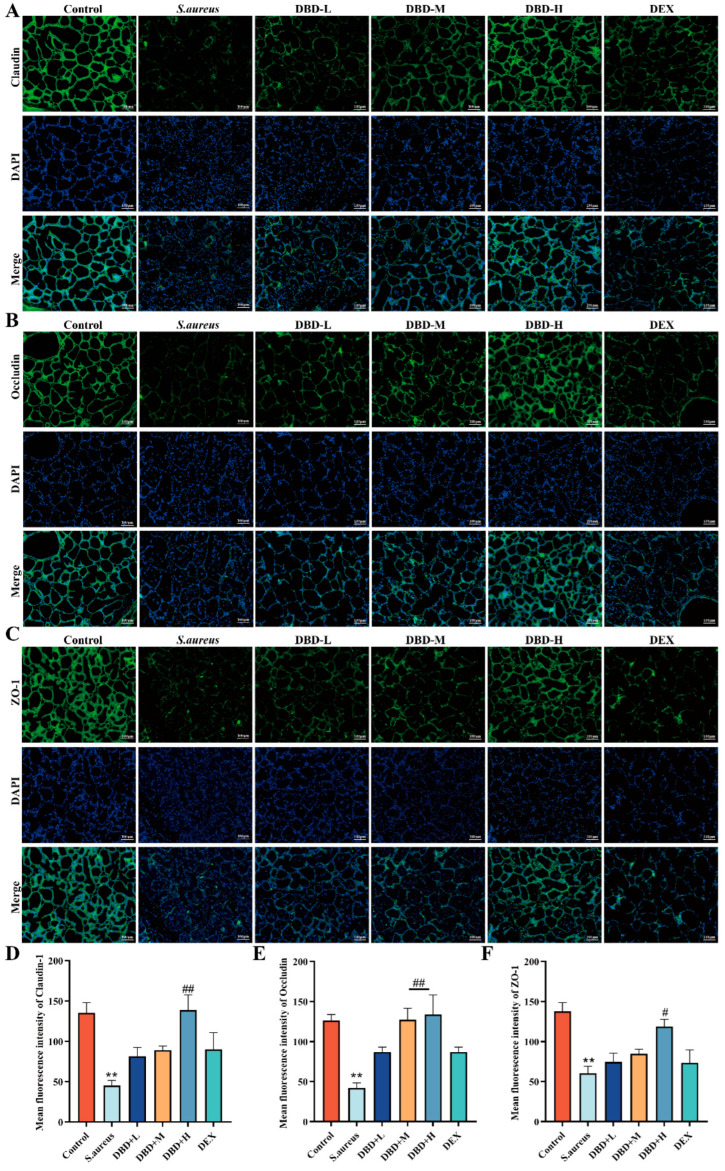
Effects of DBD on tight junction structures in mammary tissue of mice with *S. aureus*-induced mastitis. (**A**–**C**) Immunofluorescence staining (blue: DAPI; green: claudin/occludin/ZO-1; Merge: merged image) showing the effects of DBD on the expression and localization of claudin, occludin, and ZO-1 in mammary tissue (Scale bar = 100 μm). (**D**–**F**) Quantification of the mean fluorescence intensity of claudin, occludin, and ZO-1. (n = 3/group) ** *p* < 0.01 vs. the control group; ^#^ *p* < 0.05, ^##^ *p* < 0.01 vs. the *S. aureus* group.

Impairment of tight junction integrity can lead to dysfunction of the blood–milk barrier. To evaluate the influence of DBD on this barrier in murine mastitis, the ultrastructural morphology of mammary epithelial tight junctions was examined by transmission electron microscopy. Relative to the control animals, tissue from the *S. aureus*-infected group displayed substantial disruption of junctional integrity, including visible gaps. DBD administration considerably attenuated these pathological alterations. Mice receiving the high DBD dosage showed clearly defined and largely continuous tight junction structures, closely resembling those in healthy controls (Figure 7A). These observations support a dose-dependent restorative role of DBD on the blood–milk barrier. At the molecular level, Western blot analysis revealed that infection significantly suppressed mammary tissue expression of claudin-1, occludin, and ZO-1 relative to the control group (*p* < 0.05). In contrast, DBD treatment significantly upregulated these proteins (*p* < 0.05; Figure 7B,C). Immunofluorescence staining corroborated these results, demonstrating that DBD promoted the restoration of normal expression and cellular distribution for all three tight junction proteins (Figure 8A–C).

### 3.6. DBD Suppresses NF-κB/NLRP3 Signaling Pathway Activation in S. aureus-Induced Mastitis

**Figure 9:**
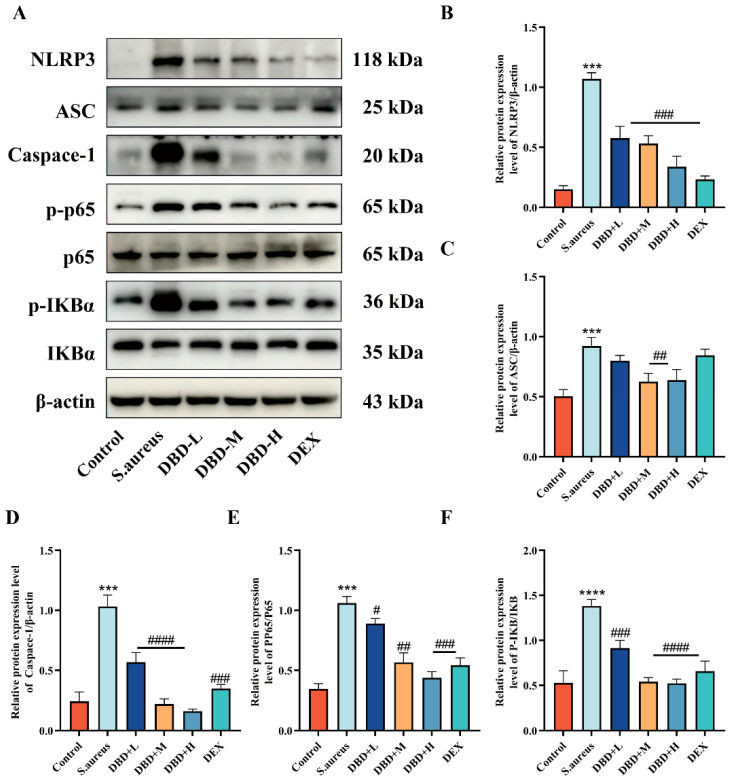
DBD inhibits the activation of NF-κB/NLRP3 in *S. aureus*-induced mastitis. (**A**) Protein expression levels of NF-κB and NLRP3 pathway components were detected by Western blot. (**B**–**E**) Relative intensity of NLRP3, ASC, Caspase-1, p-p65, and p-IκBα. (n = 3/group) *** *p* < 0.001, **** *p* < 0.0001 vs. control group; ^#^ *p* < 0.05, ^##^ *p* < 0.01, ^###^ *p* < 0.001, ^####^ *p* < 0.0001 vs. *S. aureus* group.

As shown by Western blot, phosphorylation levels of the NF-κB signaling components p65 and IκBα in mammary tissue were markedly increased following *S. aureus* infection compared with those in the control group. (*p* < 0.01), reflecting substantial activation of this pathway. DBD administration effectively suppressed IκBα phospho-degradation and reduced p65 phosphorylation in a concentration-dependent manner (*p* < 0.01), indicating that DBD attenuates NF-κB signaling through IκBα stabilization. In the NLRP3 inflammasome pathway, infection markedly increased the expression levels of NLRP3, ASC, and cleaved caspase-1. (*p* < 0.01), and promoted the interaction between NLRP3 and ASC, confirming inflammasome activation. DBD treatment markedly downregulated the levels of these proteins and interfered with NLRP3–ASC complex assembly (Figure 9A,F). Moreover, DBD concurrently diminished the secretion of cytokines associated with NF-κB (e.g., TNF-α and IL-6) and those dependent on NLRP3 (e.g., IL-1β), underscoring its dual inhibitory effect on both inflammatory signaling axes.

### 3.7. DBD Suppresses MAPK Signaling Pathway Activation in S. aureus-Induced Mastitis

**Figure 10:**
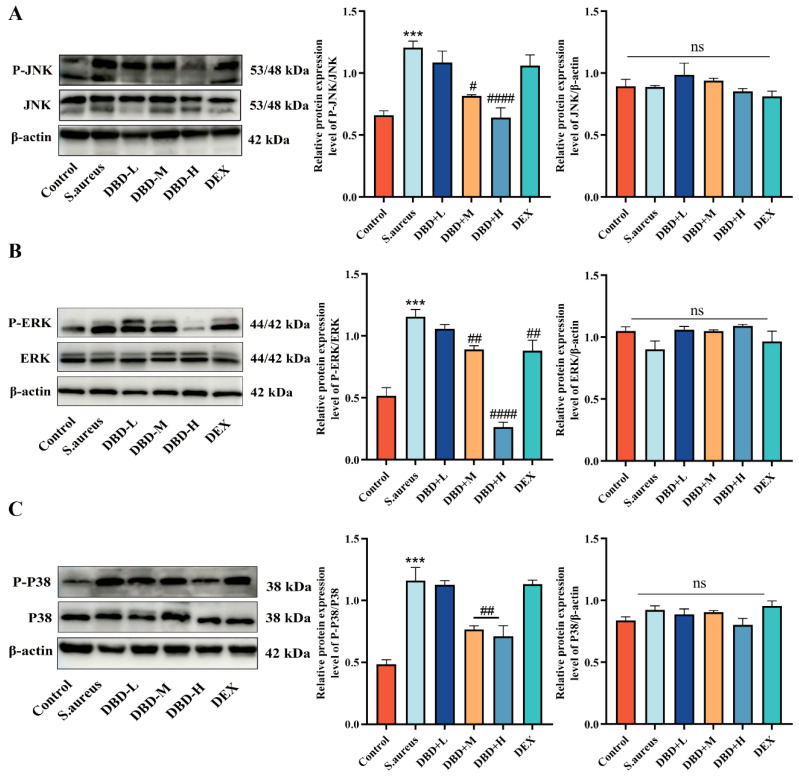
DBD inhibits the activation of MAPK in *S. aureus*-induced mastitis. (**A**–**C**) Protein expression levels of the MAPK pathway were detected by Western blot. *** *p* < 0.001 vs. control group; (n = 3/group) ^#^ *p* < 0.05, ^##^ *p* < 0.01, ^####^ *p* < 0.0001 vs. *S. aureus* group; ns, not significant.

The MAPK signaling pathway plays a vital role in inflammatory processes and has been implicated in the pathogenesis of mastitis. To determine whether the anti-inflammatory action of DBD involves modulation of this pathway, we assessed phosphorylation of its core components. The results indicated that relative to the control group, *S. aureus* infection induced a pronounced upregulation of phosphorylated JNK, ERK1/2, and p38 (*p* < 0.01). DBD treatment, however, significantly suppressed the activation of these proteins in a dose-dependent fashion (*p* < 0.01). These data suggest that MAPK signaling contributes to the progression of *S. aureus*-induced mastitis and that DBD mitigates mammary inflammation by restraining phosphorylation of JNK, ERK1/2, and p38, thereby attenuating signal transduction. This inhibitory activity complements the suppressive effects of DBD on the NF-κB and NLRP3 pathways, collectively reducing tissue injury (Figure 10A–C).

## 4. Discussion

Danggui Buxue Decoction (DBD), a classical herbal preparation composed of *Astragalus mongholicus* and *Angelica sinensis* in a 5:1 ratio, is widely recognized for its functions to tonify qi and enrich blood. Its active components—including polysaccharides, saponins, flavonoids, and volatile oils—contribute to diverse pharmacological activities such as anti-inflammation, antioxidative stress, immune regulation, and improved microcirculation [31]. In this research, we systematically evaluated the protective role of DBD in mitigating *S. aureus*-induced mastitis in mice and explored the underlying mechanisms. Our findings indicate that DBD markedly reduced pathological damage and inflammatory responses in infected mammary tissue. Mechanistically, DBD suppressed the activation of the NF-κB/NLRP3 and MAPK signaling pathways by inhibiting the phosphorylation of core proteins. Moreover, DBD administration modulated the composition of the gut microbiota, enriching beneficial genera such as *Lactobacillus* while decreasing opportunistic pathogens like *Escherichia*-*Shigella*, which further contributed to inflammation resolution. Collectively, these results reveal the multi-target regulatory potential of DBD in mitigating mastitis.

Mastitis represents a widespread inflammatory condition affecting mammals, leading to substantial economic impacts on dairy production and livestock farming globally [2]. Infection with *S. aureus* triggers a multifactorial inflammatory disorder characterized by aberrant immune activation, exaggerated inflammatory signaling, and disturbances in gut microbial ecology [10,32,33]. Typical histopathological manifestations include mammary hyperemia, edema, structural disorganization of alveoli, and substantial accumulation of inflammatory cells—particularly neutrophils [34]. In the current investigation, DBD administration notably ameliorated mammary tissue congestion and limited neutrophil infiltration. Myeloperoxidase (MPO), an enzymatic marker reflecting neutrophil activity, serves as a critical parameter for evaluating mastitis severity [35]. Our data revealed that DBD strongly suppressed MPO activity in mammary tissue. *S. aureus* initiates innate immune responses through engagement of pattern recognition receptors such as TLR4, stimulating macrophages and other immune cells to release key pro-inflammatory cytokines such as TNF-α and IL-6, chemokines, and reactive oxygen species, thereby propagating an inflammatory cascade [17]. Previous reports have consistently shown that *S. aureus* infection upregulates these inflammatory mediators and aggravates tissue injury [36]. The present work demonstrates that DBD effectively curtails the production of these factors, mitigating *S. aureus*-induced mammary inflammation, and supports its potential utility in mastitis management.

The interaction between gut microbiota and mastitis has been extensively studied in dairy cow models [37]; however, research on TCM interventions in murine models remains limited [38]. As highlighted by Volzing et al. [39], growing evidence supports the involvement of gut microbiota in disease prevention. For example, previous studies have reported that some probiotic taxa, such as Bifidobacterium, can inhibit pathogen growth through the secretion of antimicrobial compounds. Additionally, microbiota-derived metabolites, including short-chain fatty acids (SCFAs), have been reported to help maintain intestinal epithelial barrier integrity and influence immune homeostasis in distant organs such as the lungs and mammary glands through systemic circulation [40].

In the present study, we evaluated the effects of DBD on *S. aureus*-induced mastitis in a murine model. DBD treatment was associated with increased gut bacterial α-diversity, as reflected by changes in the Chao1, ACE, Shannon, and Simpson indices compared with the model group. In addition, PCoA and PLS-DA analyses revealed distinct clustering among the control, model, and DBD groups, indicating that DBD treatment was associated with alterations in the overall gut microbial community structure. At lower taxonomic levels, DBD treatment was associated with increased relative abundances of Bacteroides, Candidatus Arthromitus, Lactobacillales, and Muribaculaceae, together with a decreased relative abundance of Escherichia-Shigella. These changes suggest that DBD treatment may modulate gut microbial composition in mice with *S. aureus*-induced mastitis. Nevertheless, the specific functional relevance of these altered taxa remains to be clarified. These observations are partly consistent with the previously reported effects of Astragalus polysaccharides on gut microbial communities [41], although the specific DBD components responsible for these microbiota alterations remain to be clarified. Correlation analysis further suggested that some taxa enriched in the model group, such as Escherichia-Shigella, were positively associated with inflammatory parameters, whereas several taxa altered after DBD treatment showed opposite trends. However, these associations do not establish causality. Although causality was not established in the present study, previous evidence suggests that gut microbiota alterations may be linked to mastitis-related inflammatory responses through several possible pathways. For instance, some Lactobacillus strains have been reported to produce short-chain fatty acids (SCFAs), which can suppress NF-κB activation and reduce pro-inflammatory cytokine production in experimental settings [42,43]. Furthermore, gut dysbiosis may promote intestinal barrier dysfunction and facilitate the translocation of microbial products, such as lipopolysaccharide (LPS), into the circulation, thereby contributing to systemic inflammatory responses [44]. Based on these findings, we speculate that DBD-associated microbiota alterations may be related to its anti-inflammatory effects and protection of the blood–milk barrier. However, intestinal barrier function, circulating endotoxin levels, SCFA concentrations, and microbiota-dependent causality were not directly assessed in this study and require further investigation.

In the mammary glands of mammals, the blood–milk barrier acts as a pivotal structural element, fulfilling vital defensive functions and sustaining homeostatic physiological activities. Its functional competence is predominantly dependent on the integrity of tight junctions (TJs) formed between mammary epithelial cells [45]. Numerous prior investigations have demonstrated that mastitis is accompanied by aberrant immune cell infiltration, which not only perturbs the structural integrity of tight junctions (TJs) but also reduces the expression of TJ-related proteins—ultimately impairing the functional competence of the blood–milk barrier. This barrier dysfunction elevates the mammary gland’s vulnerability to pathogenic bacterial colonization, aggravates the extent of tissue injury, and induces the depletion of nutritive components in milk, thereby creating a vicious cycle that further amplifies the inflammatory cascade [46]. Accordingly, the preservation of TJ integrity is indispensable for both sustaining blood–milk barrier functionality and alleviating inflammatory reactions. The current study aimed to explore whether DBD possesses the capacity to mitigate blood–milk barrier impairment induced by mastitis. Experimental data demonstrated that in an *S. aureus*-mediated mastitis model, DBD facilitated the recovery of barrier integrity through enhancing the expression of TJ proteins and restoring their normal cellular distribution—thereby exerting a potent inhibitory effect on the inflammatory process.

Inflammatory responses induced by *S. aureus* involve the coordinated activation of multiple signaling cascades, with the transcription factor NF-κB acting as a critical regulator of inflammation-related genes, including cytokines and chemokines [47]. Under basal conditions, NF-κB p65 is retained in the cytoplasm through its interaction with IκBα [48]. Upon *S. aureus* infection, phosphorylation of the upstream kinase AKT promotes IκBα degradation, promoting the translocation of the NF-κB p65 subunit into the nucleus and the subsequent transcriptional activation of major pro-inflammatory mediators [49]. The NLRP3 inflammasome—a multimolecular complex consisting of NLRP3, ASC, and caspase-1—serves a critical function in innate immunity by facilitating caspase-1-governed maturation and extracellular release of the pro-inflammatory cytokines IL-1β and IL-18 [50]. NF-κB signaling is known to induce NLRP3 inflammasome assembly and enhance pro-inflammatory cytokine production [5]. Conversely, NLRP3 activation can further amplify NF-κB and MAPK signaling, creating a pro-inflammatory feedback loop [51]. Given the established involvement of the NLRP3 inflammasome in *S. aureus*-induced inflammation [52], we investigated the modulatory effect of DBD on this pathway. Our findings demonstrate that DBD markedly attenuates *S. aureus*-induced NLRP3 inflammasome activation, thereby mitigating inflammatory cascades. Targeting NLRP3 may thus represent a promising therapeutic approach for the management of *S. aureus* mastitis.

The MAPK signaling cascade, which comprises serine/threonine kinases such as ERK1/2, JNK, and p38, plays a critical role in regulating inflammatory responses and apoptotic pathways [53]. MAPK acts upstream of NF-κB and modulates its transcriptional activity [54]. Phosphorylation of MAPK components can be induced by stimuli such as LPS [55]. The present study illustrated that DBD significantly suppressed the expression of inflammatory mediators in mammary tissue by specifically reducing MAPK phosphorylation, hence alleviating mammary inflammation.

Collectively, DBD exerts notable therapeutic effects on mastitis triggered by Staphylococcus aureus, achieved by suppressing inflammatory signaling cascades and reshaping the composition of intestinal flora. Mechanistic analyses reveal that its anti-inflammatory actions are mediated through inhibition of the hyperactivated NF-κB/NLRP3 and MAPK signaling axes. These findings offer novel perspectives on mastitis prophylaxis and highlight the therapeutic capacity of DBD—a classic traditional Chinese medicine formula used for qi replenishment and blood nourishment—which operates via multi-component, multi-target synergy. Specifically, it disrupts key inflammatory signaling pathways and promotes systemic immune homeostasis through gut microbiota remodeling, underscoring its potential as an integrated intervention for inflammatory pathologies.

## 5. Limitations

Nevertheless, several limitations should be acknowledged. First, the sample size in this study was relatively small, and larger studies are needed to confirm the present findings. Second, although animals were randomly assigned to the experimental groups, outcome measurements and data analyses were were conducted without blinding, which may introduce potential bias during data collection. Third, because this study was based on a murine model, further studies are required to determine the clinical relevance of these findings. In addition, the microbiota analysis was mainly associative, and the proposed microbial mechanisms remain speculative. Although DBD treatment was associated with changes in gut microbiota composition, no direct causal relationship was established between specific microbial alterations and the protective effects against *S. aureus*-induced mammary inflammation. Furthermore, 16S rRNA sequencing provides only limited functional insight and cannot fully reflect microbial metabolic activity or host–microbiota interactions. Future studies using fecal microbiota transplantation, antibiotic depletion, metagenomics, metabolomics, and targeted functional experiments are warranted to clarify the causal contribution of gut microbiota to the protective effects of DBD.

## 6. Conclusions

In conclusion, preventive administration of DBD alleviated S. aureus-induced mammary inflammatory injury in mice, possibly by regulating inflammatory signaling pathways and modulating gut microbiota composition. These findings indicate that DBD exerts protective effects in this murine mastitis model and warrant further investigation to evaluate its therapeutic efficacy after mastitis onset, safety, pharmacokinetics, milk residue profile, and applicability in dairy cows.

## Figures and Tables

**Table 1:** Primers sequences for qRT-PCR.

Gene	Sequence (5′ → 3′)	Length, bp
*TNF-α*	F:ACGGCATGGATCTCAAAGAC	116
	R:GTGGGTGAGGAGCACGTAGT	
*IL-1β*	F:TGCCACCTTTTGACAGTGATG	136
	R:ATGTGCTGCTGCGAGATTTG	
*IL-6*	F:CCCCAATTTCCAATGCTCTCC	141
*β-actin*	R:CGCACTAGGTTTGCCGAGTA	
	F:CACTGTCGAGTCGCGTCC	146
	R:TCATCCATGGCGAACTGGTG	

## Data Availability

The original contributions presented in this study are included in the article/Supplementary Material. Further inquiries can be directed to the corresponding authors.

## Supplementary Materials

The following supporting information can be downloaded at: https://www.mdpi.com/article/10.3390/vetsci13070613/s1, Figure S1. HPLC fingerprint chromatograms of 11 batches of DGBXT samples; Table S1. Linearity parameters of five bioactive components of DGBXT; Table S2. Similarity evaluation results of 11 batches of DGBXT samples; Table S3. Content determination results of five target components in DGBXT.

## Abbreviations

The following abbreviations are used in this manuscript:

DBD	Danggui Buxue Decoction
*S. aureus*	*Staphylococcus aureus*
ELISA	Enzyme-linked immunosorbent assay
MPO	Myeloperoxidase
TNF-α	Tumor Necrosis Factor-alpha
IL-6	Interleukin-6
IL-1β	Interleukin-1 beta
COX-2	Cyclooxygenase-2
iNOS	Inducible nitric oxide synthase
qRT-PCR	Quantitative reverse transcription polymerase chain reaction
PCoA	Principal Coordinates Analysis
PLS-DA	Partial least squares discriminant analysis.
