# Supplementary material for: Danggui Buxue Decoction Attenuates Staphylococcus aureus-Induced Mastitis in Mice Associated with Gut Microbiota Remodeling, Blood–Milk Barrier Protection, and Inflammatory Suppression
Source: Vet Sci. 2026 Jun 25;13(7):613. doi: 10.3390/vetsci13070613 (PMC13417064; doi:10.3390/vetsci13070613)
Supplement: Supplementary file 1 [file vetsci-13-00613-s001.zip › vetsci-4316763-Supplementary Materials.pdf]

Supplementary Materials:

Calibration curves were constructed by plotting peak areas against the corresponding concentrations of the five reference compounds. The regression equations, correlation coefficients, and linear ranges are listed in Table S1.

Table S1 Linearity parameters of five bioactive components of DGBXT

Ingredient	Linear equation	R^2	Linear range（mg·mL^{-1}）
calycosin-7-O-glucoside	y=40287x+2.5298	0.9995	0.0030 ~ 0.0962
chlorogenic acid	y=23517x−47.182	0.9995	0.0022 ~ 0.0692
ononin	y=20315x+11.157	1	0.0036 ~ 0.1154
ferulic acid	y=66632x−4.6454	0.999	0.0019 ~ 0.0615
formononetin	y=4460.8x+2.8857	0.9997	0.0048 ~ 0.1538

Note: Y: peak area; X: concentration（mg·mL^{-1}）

Eleven batches of DGBXT samples were prepared and analyzed under the chromatographic conditions described in Section 2.5. The chromatograms were imported into the Similarity Evaluation System for Chromatographic Fingerprint of Traditional Chinese Medicine (version 2012.130723). S1 was used as the reference chromatogram, the time window was set to 0.1, and the reference chromatogram was generated using the median method. Full-spectrum peak matching was performed with multipoint correction, and the similarity values were calculated. The similarities between the chromatograms of the 11 DGBXT batches and the reference chromatogram were all greater than 0.98, indicating good consistency among batches. The average contents of calycosin-7-glucoside, chlorogenic acid, ononin, ferulic acid, and formononetin were 0.0080, 0.0058, 0.0062, 0.0099, and 0.0957 mg·mL^{-1}, respectively. The similarity results, fingerprint chromatograms, and content determination results are shown in Fig. 1, Table 2, and Table 3, respectively.

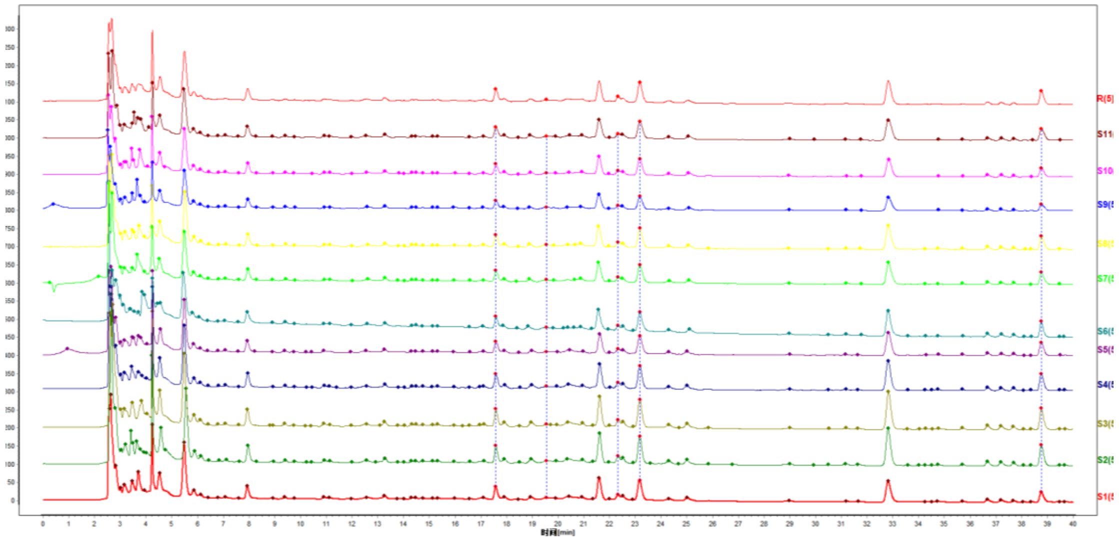

Figure S1. HPLC fingerprint chromatograms of 11 batches of DGBXT samples. S1–S11 correspond to the 11 sample batches, and R(S) represents the reference common chromatogram established by the median method.

Vertical dotted lines indicate the characteristic peaks of the five quantitative marker compounds (calycosin-7-O-glucoside, chlorogenic acid, ononin, ferulic acid, and formononetin).

Table S2. Similarity evaluation results of 11 batches of DGBXT samples

	**S1**	**S2**	**S3**	**S4**	**S5**	**S6**	**S7**	**S8**	**S9**	**S10**	**S11**	**R**
S1	1	0.985	0.984	0.989	0.991	0.993	0.994	0.994	0.999	0.999	0.992	0.994
S2	0.985	1	1	0.999	0.998	0.997	0.996	0.997	0.979	0.985	0.998	0.997
S3	0.984	1	1	0.999	0.998	0.997	0.996	0.996	0.979	0.985	0.998	0.997
S4	0.989	0.999	0.999	1	0.999	0.998	0.998	0.998	0.985	0.99	0.999	0.999
S5	0.991	0.998	0.998	0.999	1	0.999	0.999	1	0.988	0.993	0.998	0.999
S6	0.993	0.997	0.997	0.998	0.999	1	1	1	0.991	0.995	0.999	1
S7	0.994	0.996	0.996	0.998	0.999	1	1	1	0.992	0.996	0.998	1
S8	0.994	0.997	0.996	0.998	1	1	1	1	0.991	0.995	0.998	1
S9	0.999	0.979	0.979	0.985	0.988	0.991	0.992	0.991	1	0.999	0.988	0.991
S10	0.999	0.985	0.985	0.99	0.993	0.995	0.996	0.995	0.999	1	0.992	0.995
S11	0.992	0.998	0.998	0.999	0.998	0.999	0.998	0.998	0.988	0.992	1	0.999
R	0.994	0.997	0.997	0.999	0.999	1	1	1	0.991	0.995	0.999	1

Note: R, reference chromatogram generated by the median method.

Table S3 Content determination results of five target components in DGBXT

**Ingredient**	**Mean concentration (mg·mL^{-1})**	**SD (mg·mL^{-1})**
Calycosin-7-O-glucoside	0.0080	0.0014
Chlorogenic acid	0.0058	0.0023
Ononin	0.0062	0.0012
Ferulic acid	0.0099	0.0016
Formononetin	0.0957	0.0287
