# Supplementary material for: Danggui Buxue Decoction Attenuates Staphylococcus aureus-Induced Mastitis in Mice Associated with Gut Microbiota Remodeling, Blood–Milk Barrier Protection, and Inflammatory Suppression
Source: Vet Sci. 2026 Jun 25;13(7):613. doi: 10.3390/vetsci13070613 (PMC13417064; doi:10.3390/vetsci13070613)
Supplement: Supplementary file 1 [file vetsci-13-00613-s001.zip › WB/Original Images for Blots.pdf]

Original Images for Blots

Figure 3. DBD alleviates *S. aureus*-induced mastitis in mice

(G–I) Protein levels of COX-2 and iNOS detected by Western blot, with relative intensities of COX-2 and iNOS quantified.

<table>
<tr><th>Protein</th><th>Blot
| Con | S. aureus | DBD-L | DBD-M | DBD-H | DEX |</th><th>Marker</th></tr>
<tr><td>COX-2
(69 kDa)</td><td>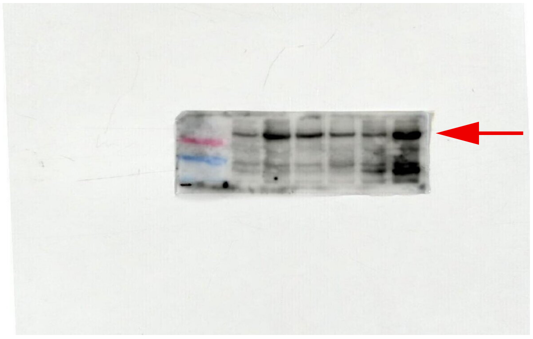</td><td>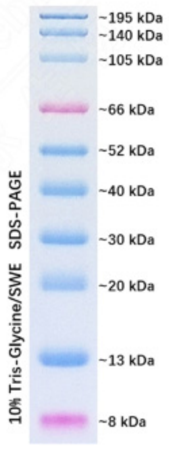
</td></tr>
<tr><td>iNOS
(110 kDa)</td><td>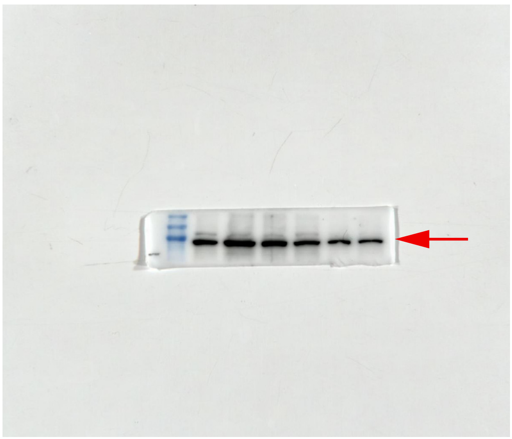</td><td>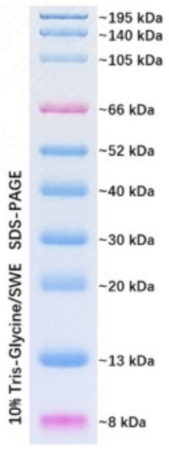
</td></tr>
<tr><td>GAPDH
(37 kDa)</td><td>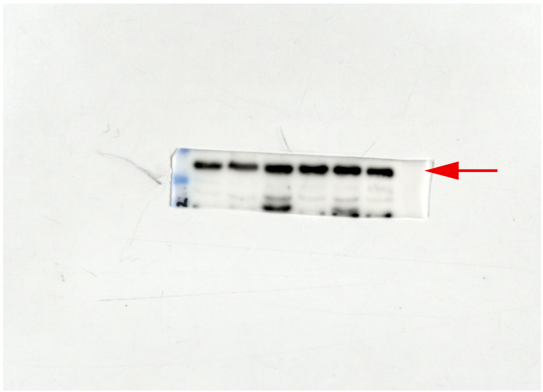</td><td>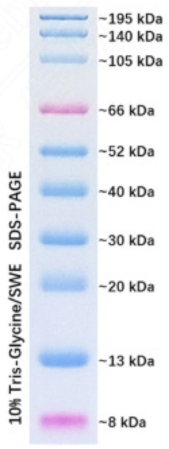
</td></tr>
</table>

Figure 7. Effect of DBD on *S. aureus*-induced tight junction injury in mouse mammary tissue.

(B–D) Expression of tight junction proteins (claudin-1, occludin, and ZO-1) in mammary tissue detected by Western blot.

Protein	Blot<br>| Con | *S. aureus* | DBD-L | DBD-M | DBD-H | DEX |	Marker
Claudin-1<br>(22kDa)	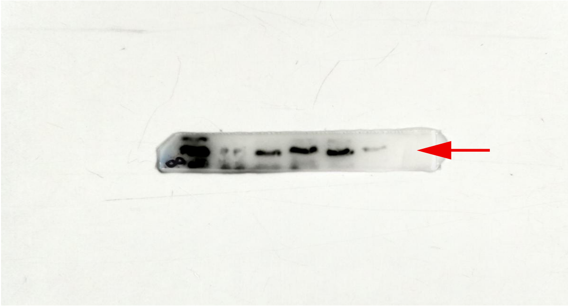	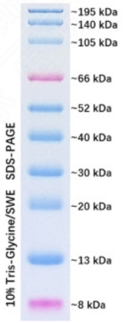
β-actin<br>(42 kDa)	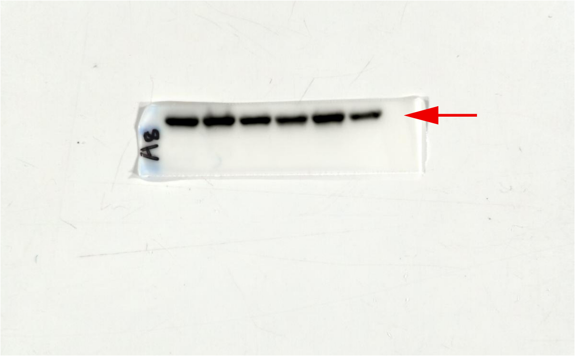	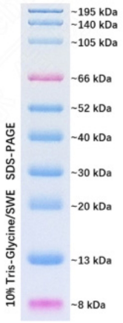
Occludin<br>(65 kDa)	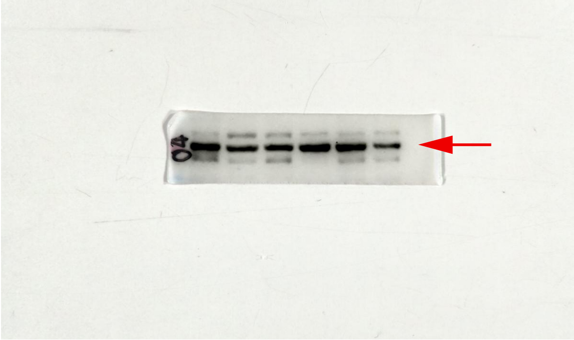	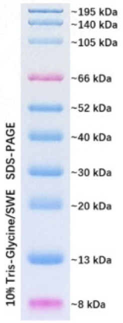
β-actin<br>(42 kDa)	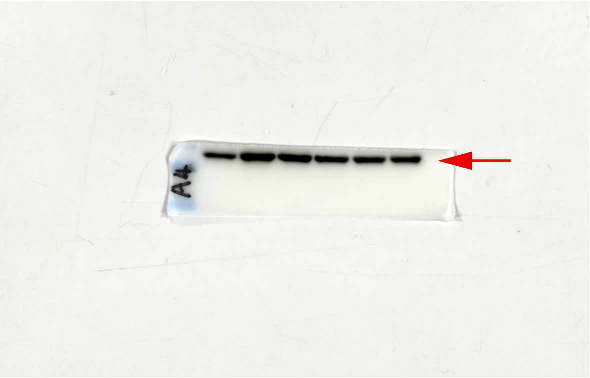	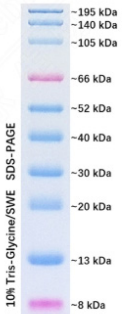

<table>
<tr><th>Protein</th><th>Blot
| Con | S. aureus | DBD-L | DBD-M | DBD-H | DEX |</th><th>Marker</th></tr>
<tr><td>ZO-1
(~120 kDa)</td><td>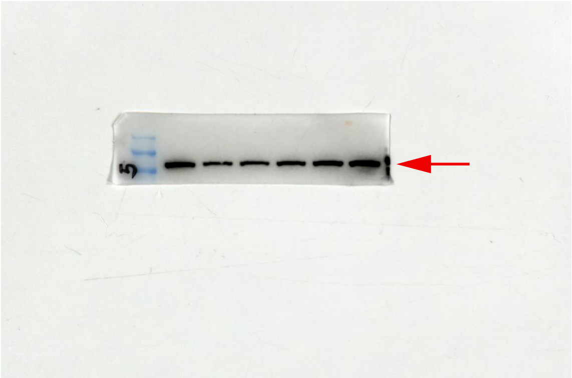</td><td>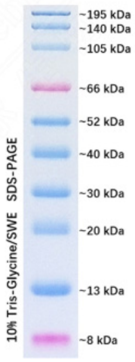
</td></tr>
<tr><td>β-actin
(42 kDa)</td><td>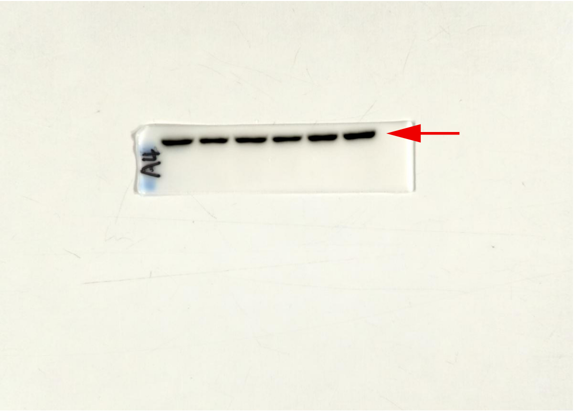</td><td>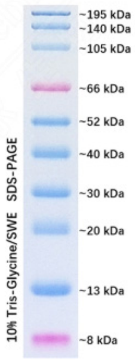
</td></tr>
</table>

Figure 9. DBD inhibits the activation of NF-κB/NLRP3 in *S. aureus*-induced mastitis. (A) Protein expression levels of NF-κB and NLRP3 pathway components were detected by Western blot.

**Protein**	**Blot**<br>**| Con | *S. aureus* | DBD-L | DBD-M | DBD-H | DEX |**	**Marker**
NLRP3<br>(118 kDa)	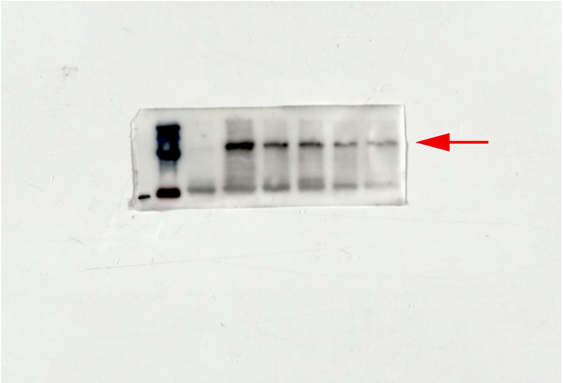	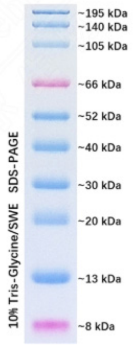
ASC<br>(25 kDa)	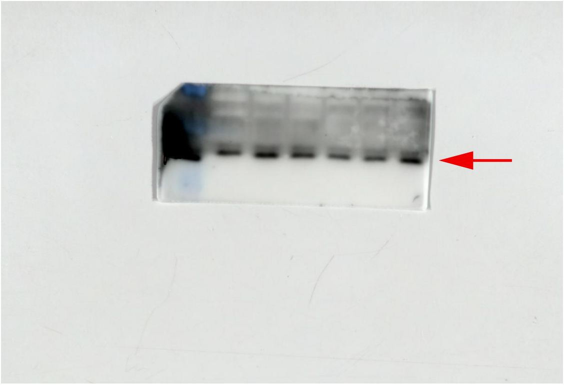	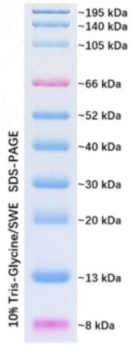
CASP1<br>(20 kDa)	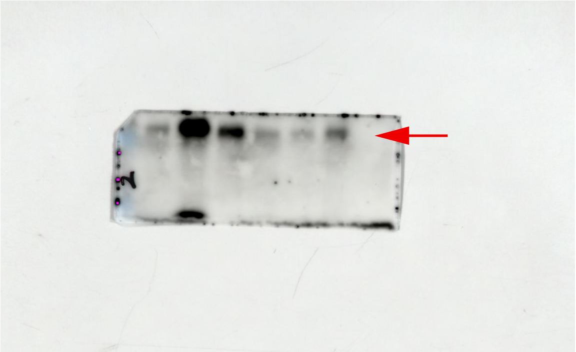	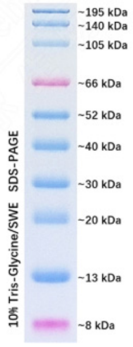
P-P65<br>(65 kDa)	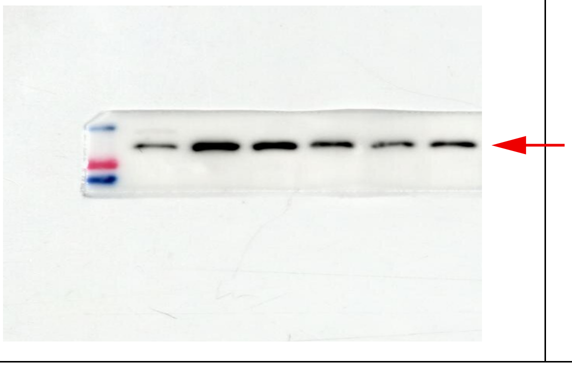	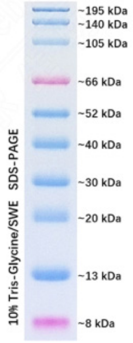

**Protein**	**Blot**<br>**| Con | *S. aureus* | DBD-L | DBD-M | DBD-H | DEX |**	**Marker**
P65<br>(65 kDa)	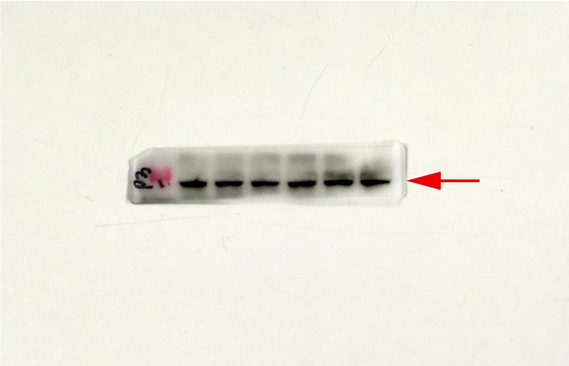	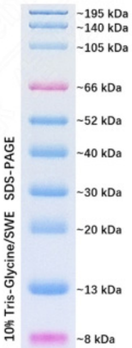
P-IKB<br>(36 kDa)	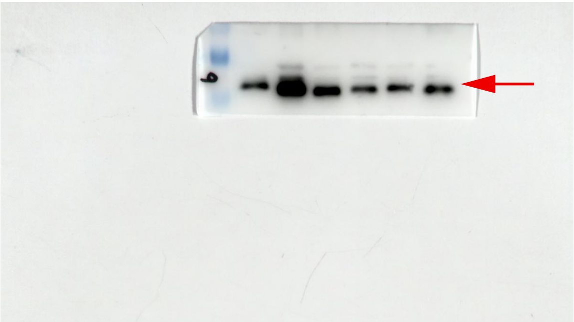	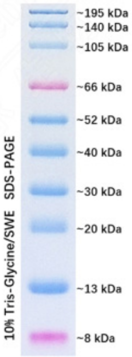
IKB<br>(35 kDa)	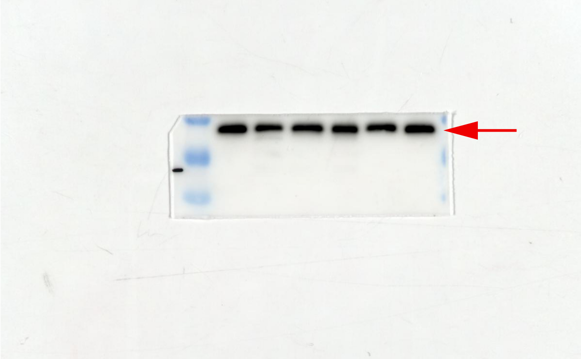	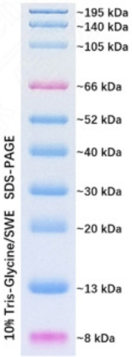
β-actin<br>(42 kDa)	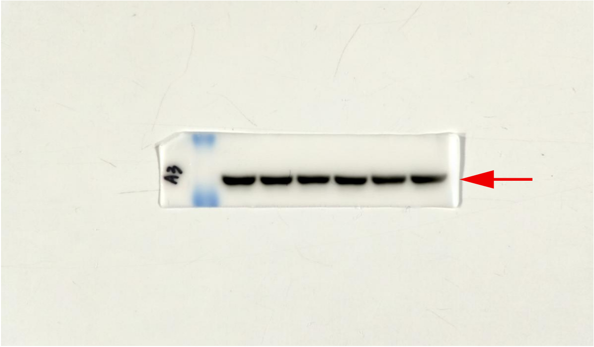	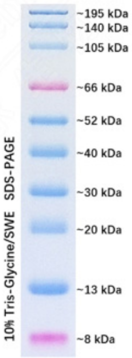

Figure 10. DBD inhibits the activation of MAPK in *S. aureus*-induced mastitis. (A–C) Protein expression levels of the MAPK pathway were detected by Western blot.

Protein	Blot<br>| Con | *S. aureus* | DBD-L | DBD-M | DBD-H | DEX |	Marker
P-JNK<br>(53/48 kDa)	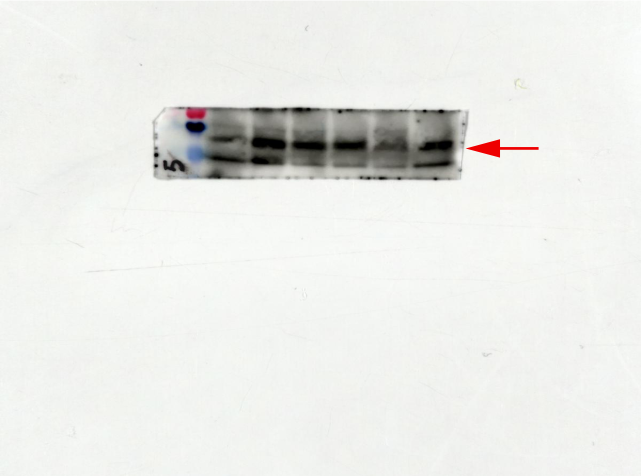	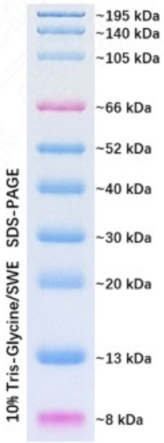
JNK<br>(53/48 kDa)	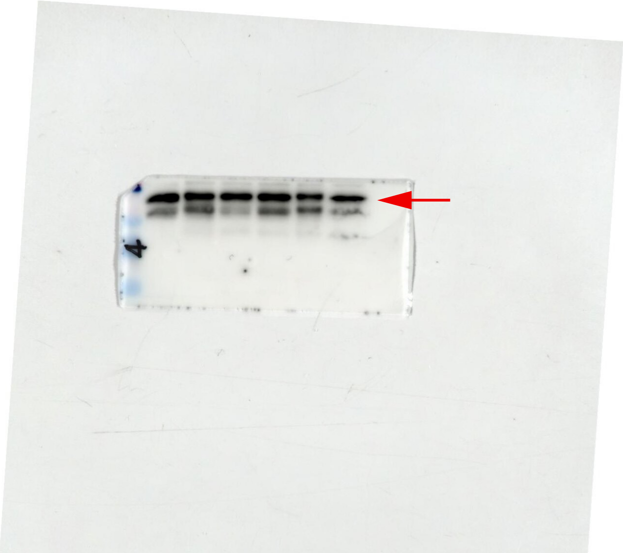	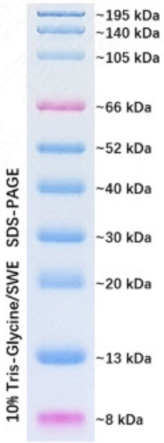
β-actin<br>(42 kDa)	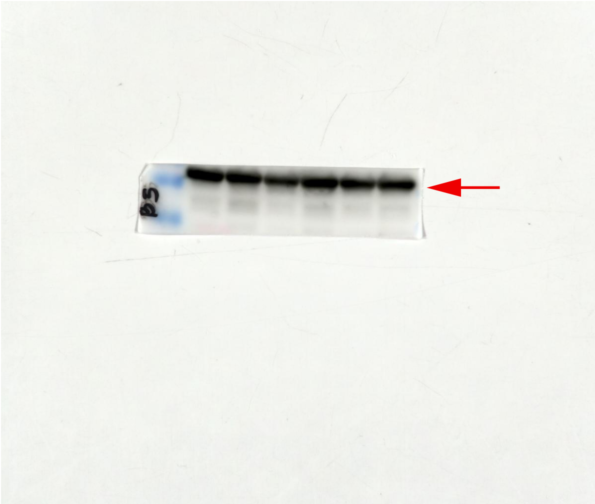	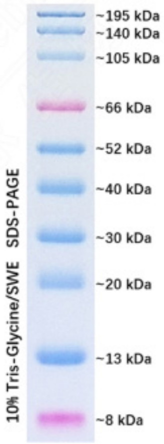

<table>
<tr><th>Protein</th><th>Blot
| Con | S. aureus | DBD-L | DBD-M | DBD-H | DEX |</th><th>Marker</th></tr>
<tr><td>P-ERK
(44/42 kDa)</td><td>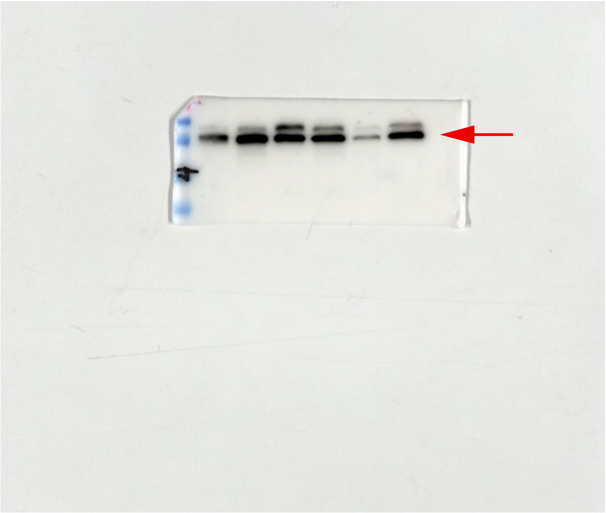</td><td>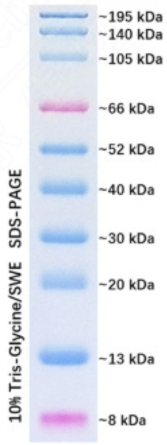
</td></tr>
<tr><td>P-ERK
(44/42 kDa)</td><td>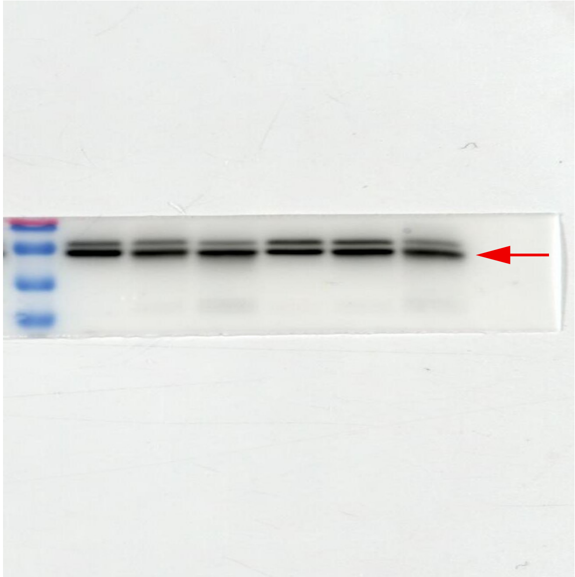</td><td>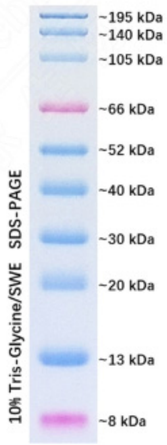
</td></tr>
<tr><td>β-actin
(42 kDa)</td><td>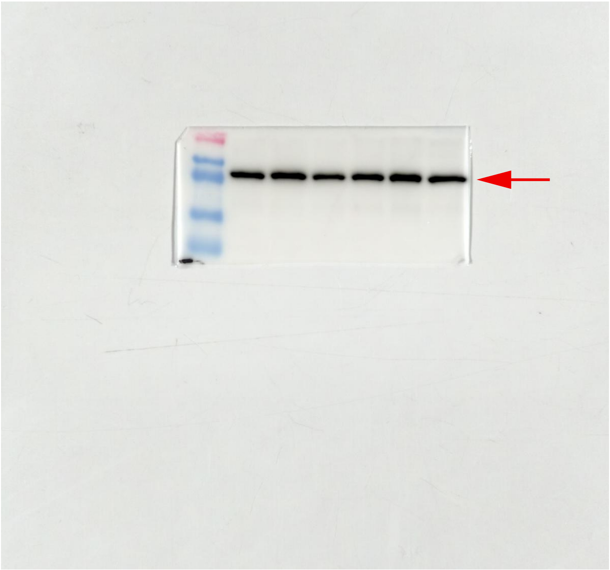</td><td>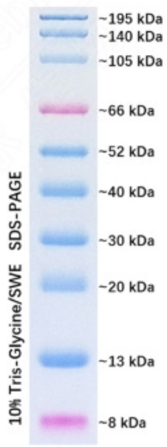
</td></tr>
</table>

<table>
<tr><th>Protein</th><th>Blot
| Con | S. aureus | DBD-L | DBD-M | DBD-H | DEX |</th><th>Marker</th></tr>
<tr><td>P-P38
(38 kDa)</td><td>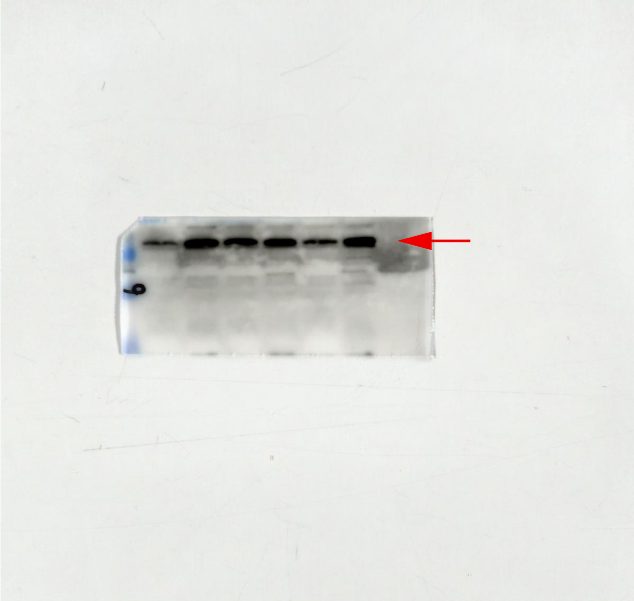</td><td>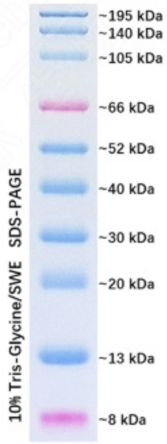
</td></tr>
<tr><td>P38
(38 kDa)</td><td>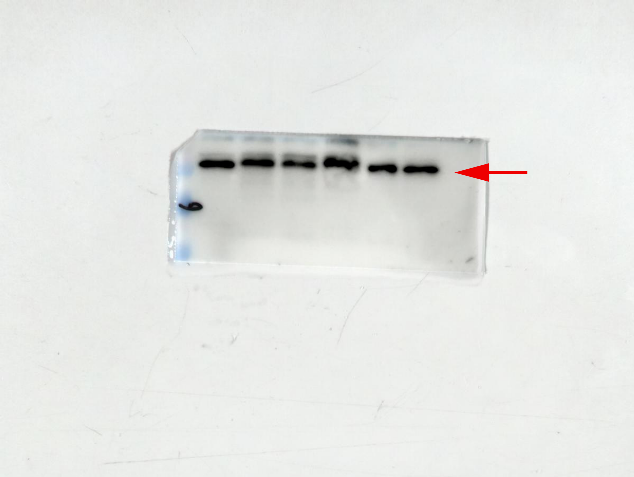</td><td>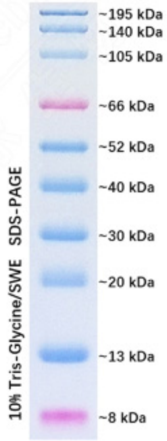
</td></tr>
<tr><td>β-actin
(42 kDa)</td><td>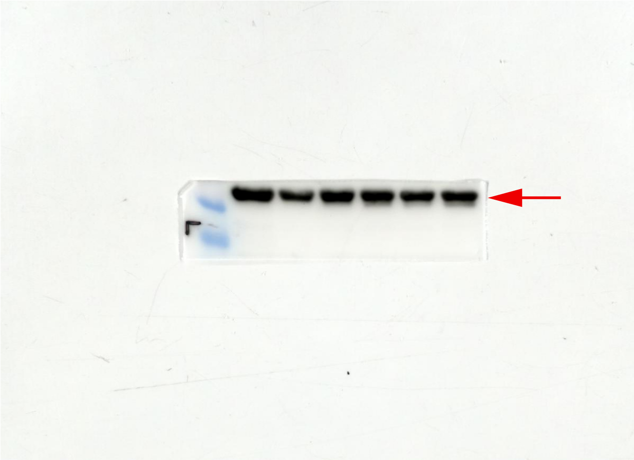</td><td>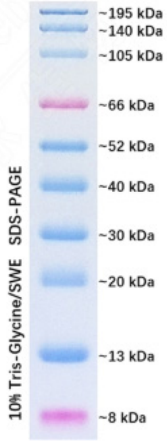
</td></tr>
</table>
