# Supplementary material for: Danggui Buxue Decoction Attenuates Staphylococcus aureus-Induced Mastitis in Mice Associated with Gut Microbiota Remodeling, Blood–Milk Barrier Protection, and Inflammatory Suppression
Source: Vet Sci. 2026 Jun 25;13(7):613. doi: 10.3390/vetsci13070613 (PMC13417064; doi:10.3390/vetsci13070613)

Figure 3. RAW Data of Western Blot

	GAPDH	COX2	
1	19199.54	9364.79	0.487761
2	18394.69	23046.08	1.252866
3	27282.47	15942.25	0.58434
4	27964.37	15150.86	0.541792
5	26933.71	13588	0.504498
6	21126.76	30629.05	1.449775
1	19834.95	9636.619	0.48584
2	18827.69	22298.95	1.18437
3	26949.64	15486.08	0.57463
4	27703.95	14033.57	0.506555
5	25214.32	13962.71	0.553761
6	22951.05	30009.59	1.307548
1	17857.54	9418.376	0.527417
2	16471.4	21703.08	1.317622
3	27052.13	14586.54	0.539201
4	27568.25	16757.4	0.607851
5	26308.13	14061	0.534474
6	19362.93	29639.76	1.530747

Control	S.aureus	DBD+L	DBD+M	DBD+H	DEX
0.487761	1.252866	0.58434	0.541792	0.504498	1.449775
0.48584	1.18437	0.57463	0.506555	0.553761	1.307548
0.527417	1.317622	0.539201	0.607851	0.571578	1.530747

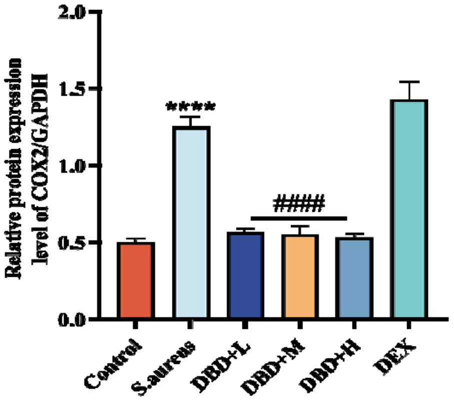

	GAPDH	INOS	
1	19199.54	19895.4	1.036243
2	18394.69	37360.13	2.031028
3	27282.47	28116.52	1.030571
4	27964.37	24301.98	0.869034
5	26933.71	14686.35	0.545278
6	21126.76	13769.59	0.651761
1	19834.95	25215.81	1.271282
2	18827.69	32252.42	1.713031
3	26949.64	25057.1	0.929775
4	27703.95	21200.03	0.765235
5	25214.32	15443.88	0.612504
6	22951.05	13558.52	0.590758
1	17857.54	19783.52	1.107852
2	16471.4	31774.25	1.929056
3	27052.13	24176.1	0.893686
4	27568.25	18893.4	0.685332
5	26308.13	13777.69	0.523705
6	19362.93	12678.35	0.654774

Control	S.aureus	DBD+L	DBD+M	DBD+H	DEX
1.0362435	2.031028	1.030571	0.869034	0.545278	0.651761
1.271282	1.713031	0.929775	0.765235	0.612504	0.590758
1.107852	1.929056	0.893686	0.685332	0.523705	0.654774

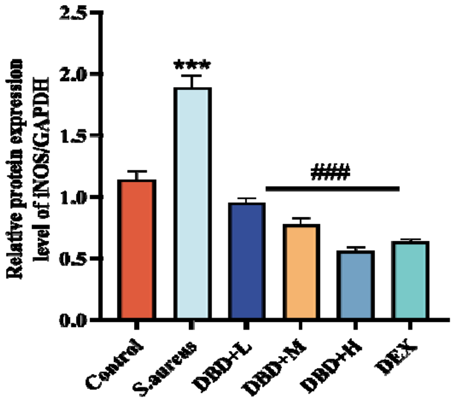

Figure 7. RAW Data of Western Blot

	β-actin	claudin1	
1	26921.03	36110.42	1.341346
2	28773.13	7713.104	0.268066
3	22196.33	16202.3	0.729954
4	21089.52	18670.76	0.88531
5	20787.28	16779.42	0.807197
6	16180.59	1805.598	0.11159
1	27163.86	32343.05	1.190665
2	28433	9506.054	0.334332
3	21958.62	11129.23	0.506827
4	20464.86	14946.93	0.730371
5	15785.57	12063.23	0.764193
6	15687.18	1945.012	0.123987
1	27457.86	33898.83	1.234577
2	28502.71	7738.225	0.271491
3	21463.62	13808.88	0.643362
4	20347.05	19937.76	0.979884
5	18600.69	17384	0.934589
6	14954.05	1927.426	0.12889

Control	S.aureus	DBD+L	DBD+M	DBD+H	DEX
1.341346	0.268066	0.729954	0.88531	0.807197	0.11159
1.190665	0.334332	0.506827	0.730371	0.764193	0.123987
1.234577	0.343637	0.562774	0.766826	0.934589	0.124153

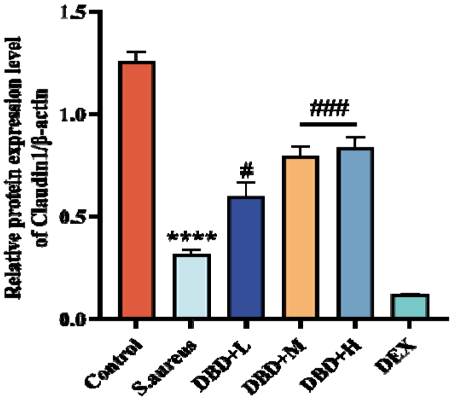

	β-actin	occludin	
1	20706.1	27650.52	1.33538
2	32288.4	15472	0.479182
3	27845.4	22021.23	0.790839
4	22985.03	24582.59	1.069504
5	21601.1	29030.05	1.343915
6	21349.23	13516.23	0.633101
1	21756.18	30591	1.406084
2	31909.13	19557.1	0.6129
3	27882.28	18577.23	0.666274
4	23641.98	23938.98	1.012562
5	21100.45	26032.1	1.233723
6	20236.23	11498.35	0.568206
1	22543.93	30014.95	1.331398
2	31003	18330.81	0.591259
3	26507.15	15541.28	0.586305
4	22563.33	19379.91	0.858912
5	20360.15	24637.64	1.210091
6	19361.71	10905.71	0.563262

Control	S.aureus	DBD+L	DBD+M	DBD+H	DEX
1.33538	0.479182	0.790839	1.069504	1.343915	0.633101
1.406084	0.6129	0.666274	1.012562	1.233723	0.568206
1.331398	0.591259	0.586305	0.858912	1.210091	0.563262

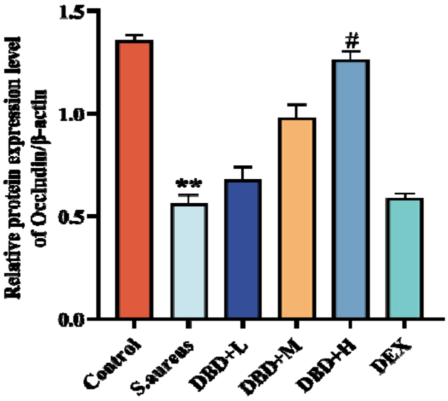

	β-actin	ZO-1	
1	28060.3	29200.95	1.04065
2	20124.13	9578.761	0.475984
3	23019.71	12013.69	0.521887
4	19643.18	14725.69	0.749659
5	24345.71	20427.1	0.839043
6	32094.05	29788.3	0.928156
1	29859.95	32014.83	1.072166
2	21472.47	9585.397	0.446404
3	22961.28	9144.74	0.398268
4	20233.4	11502.03	0.568468
5	23361.18	18003.81	0.770672
6	25650.95	27632.83	1.077263
1	26006	28709.05	1.103939
2	16480.52	8577.518	0.520464
3	17696.28	9210.861	0.520497
4	15616.74	11532.91	0.738497
5	17423.69	18582.64	1.066516
6	30985.83	28079	0.906188

Control	S.aureus	DBD+L	DBD+M	DBD+H	DEX
1.04065	0.475984	0.521887	0.749659	0.839043	0.928156
1.072166	0.446404	0.398268	0.568468	0.770672	1.077263
1.077263	0.520464	0.520497	0.738497	1.066516	0.906188

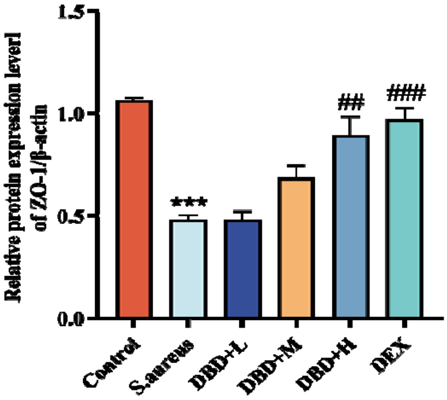

Figure 9. RAW Data of Western Blot

	β-actin	NLRP3	
1	29795.66	3641.355	0.122211
2	28161.52	28599.02	1.015536
3	27384.62	13053.05	0.476656
4	27858.45	14126.18	0.50707
5	27852.35	7861.347	0.282251
6	26275.95	6278.861	0.238958
1	30389.59	4462.477	0.146842
2	29000.23	32200.68	1.11036
3	28018.57	16286.05	0.581259
4	29099.57	14056.35	0.483043
5	28299.81	8307.225	0.293543
6	27776.49	5659.761	0.203761
1	30886.3	5644.406	0.182748
2	28991.52	31506.56	1.086751
3	28163.86	18971.52	0.673612
4	29389.98	17765.52	0.604475
5	28143.64	12359.81	0.439169
6	28101.2	7238.589	0.25759

Control	S.aureus	DBD+L	DBD+M	DBD+H	DEX
0.122211	1.015536	0.476656	0.50707	0.282251	0.238958
0.146842	1.11036	0.581259	0.483043	0.293543	0.203761
0.182748	1.086751	0.673612	0.604475	0.439169	0.25759

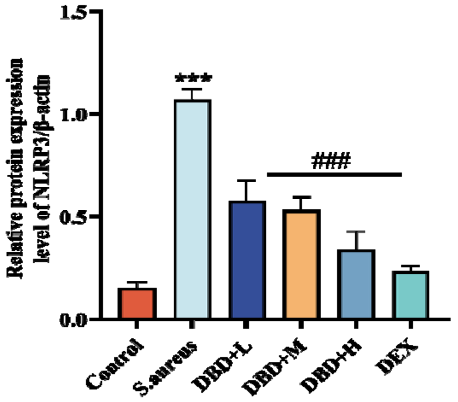

	β-actin	ASC	
1	29795.66	13368.42	0.44867
2	28161.52	28072.82	0.99685
3	27384.62	23131.97	0.844707
4	27858.45	19020.64	0.68276
5	27852.35	18331.35	0.658162
6	26275.95	23736.54	0.903356
1	30389.59	17001.76	0.55946
2	29000.23	24854.66	0.857051
3	28018.57	22374.92	0.798575
4	29099.57	18771.18	0.645067
5	28299.81	20254.88	0.715725
6	27776.49	22749.35	0.819014
1	30886.3	15572.3	0.504181
2	28991.52	26581.37	0.916867
3	28163.86	21319.13	0.756967
4	29389.98	16100.47	0.547822
5	28143.64	15222.05	0.54087
6	28101.2	22891.05	0.814594

Control	S.aureus	DBD+L	DBD+M	DBD+H	DEX
0.44867	0.99685	0.844707	0.68276	0.658162	0.903356
0.55946	0.857051	0.798575	0.645067	0.715725	0.819014
0.504181	0.916867	0.756967	0.547822	0.54087	0.814594

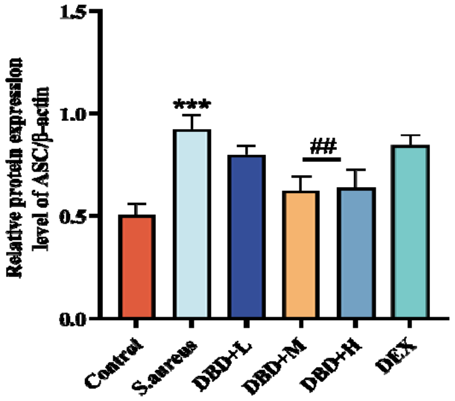

	β-actin	Caspase-1	
1	29795.66	6278.033	0.210703
2	28161.52	31831.47	1.130318
3	27384.62	17422.83	0.636227
4	27858.45	5107.326	0.183331
5	27852.35	4390.69	0.157642
6	26275.95	8569.225	0.326124
1	30389.59	5640.569	0.185609
2	29000.23	29701.49	1.024181
3	28018.57	16489.59	0.588524
4	29099.57	6077.933	0.208867
5	28299.81	4117.468	0.145495
6	27776.49	9172.518	0.330226
1	30886.3	8165.761	0.264381
2	28991.52	27233.66	0.939366
3	28163.86	13498.76	0.479294
4	29389.98	7807.104	0.265638
5	28143.64	5055.811	0.179643
6	28101.2	10931.85	0.389017

Control	S.aureus	DBD+L	DBD+M	DBD+H	DEX
0.210703	1.130318	0.636227	0.183331	0.157642	0.326124
0.185609	1.024181	0.588524	0.208867	0.145495	0.330226
0.330226	0.939366	0.479294	0.265638	0.179643	0.389017

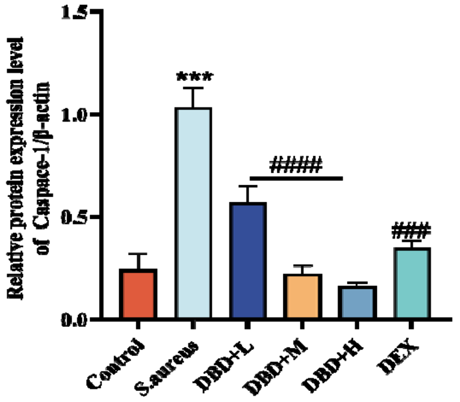

	p65	pp65	
1	25392.66	9755.418	0.384183
2	25017.81	27887.59	1.114709
3	24469.47	22951.42	0.937961
4	26094.13	16762.95	0.642403
5	27654.71	12522.95	0.452833
6	23580.54	14441.71	0.612442
1	26426.18	9355.418	0.354021
2	27745.64	27856.59	1.003999
3	24317.64	20884.42	0.858818
4	26618.3	15280	0.574041
5	26708.47	10211.2	0.382321
6	22820.44	11907.71	0.5218
1	29951.66	8925.539	0.297998
2	26435.4	28102.18	1.063051
3	23852.93	20842.3	0.873783
4	26144.47	12659.76	0.484223
5	25428.35	12190.61	0.47941
6	21741.15	10765.18	0.495152

Control	S.aureus	DBD+L	DBD+M	DBD+H	DEX
0.384183	1.114709	0.937961	0.642403	0.452833	0.612442
0.354021	1.003999	0.858818	0.574041	0.382321	0.5218
0.297998	1.063051	0.873783	0.484223	0.47941	0.495152

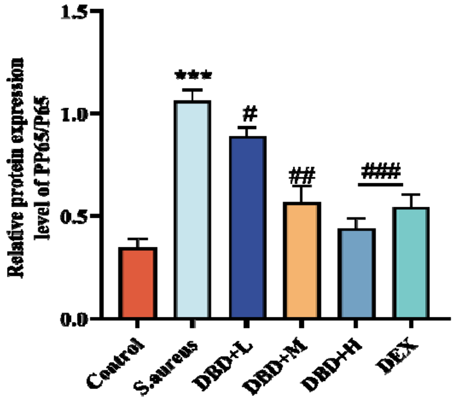

	IKB	P-IKB	
1	26328.64	10552.05	0.400782
2	20719.59	30199.86	1.457551
3	24876.47	20996.33	0.844024
4	22881.38	13458.52	0.588187
5	22773.76	13086	0.574609
6	26040.81	16725.3	0.642272
1	25558.95	13112.93	0.513047
2	19851.59	27258.81	1.37313
3	22750.05	22964.62	1.009431
4	21741.91	11590.4	0.53309
5	19731.1	9554.054	0.484213
6	23600.05	18435.32	0.781156
1	25412.88	17025.35	0.669949
2	19559.76	25783.81	1.318207
3	21546.13	19196.62	0.890955
4	21306.03	10639.15	0.499349
5	18431.35	9412.811	0.510696
6	22912.05	14303.32	0.62427

Control	S.aureus	DBD+L	DBD+M	DBD+H	DEX
0.400782	1.457551	0.844024	0.588187	0.574609	0.56547
0.513047	1.37313	1.009431	0.53309	0.484213	0.781156
0.669949	1.318207	0.890955	0.499349	0.510696	0.62427

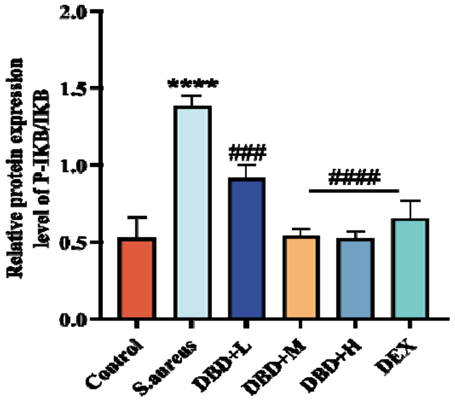

Figure 10. RAW Data of Western Blot

	jnk	p-jnk	
1	24523.18	17455.18	0.711783
2	27789.13	30712.05	1.105182
3	20420.83	25787.81	1.262819
4	29759.78	24301.52	0.816589
5	20887.88	12857.83	0.615564
6	18391.83	17609.66	0.957472
1	27468.71	18595.05	0.676954
2	26881.23	32878.93	1.223119
3	24536	25628.1	1.04451
4	28372	23601.05	0.831843
5	20487.3	10596.3	0.517213
6	19407.13	23947.95	1.233977
1	26343.23	15548.54	0.590229
2	26584.1	34206.93	1.286744
3	26622.83	25316.35	0.950926
4	29778.42	23891.05	0.802294
5	19850.59	15657.95	0.78879
6	21109.18	20869.71	0.988656

Control	S.aureus	DBD+L	DBD+M	DBD+H	DEX
0.711783	1.105182	1.262819	0.816589	0.615564	0.957472
0.676954	1.223119	1.04451	0.831843	0.517213	1.233977
0.590229	1.286744	0.950926	0.802294	0.78879	0.988656

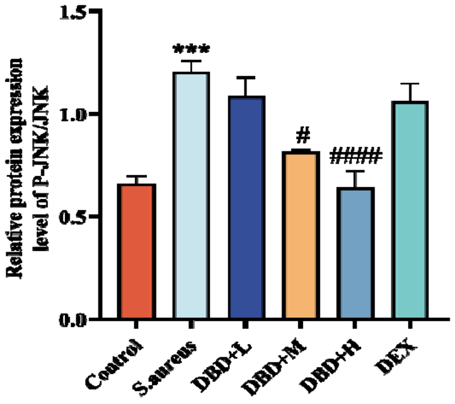

	β-actin	jnk	
1	30612.23	24523.18	0.801091
2	30534.86	27789.13	0.910079
3	25551.69	20420.83	0.799197
4	30773.62	29759.78	0.967055
5	25421.81	20887.88	0.821652
6	25177.81	18391.83	0.730478
1	27616.28	27468.71	0.994657
2	30405.93	26881.23	0.884078
3	22572.35	24536	1.086994
4	31308.4	28372	0.906211
5	22845.93	20487.3	0.896759
6	22682.18	19407.13	0.855611
1	29798.28	26343.23	0.884052
2	30517.1	26584.1	0.871121
3	24771.98	26622.83	1.074715
4	31468.28	29778.42	0.9463
5	23607.28	19850.59	0.840867
6	24746.35	21109.18	0.853022

Control	S.aureus	DBD+L	DBD+M	DBD+H	DEX
0.801091	0.910079	0.799197	0.967055	0.821652	0.730478
0.994657	0.884078	1.086994	0.906211	0.896759	0.855611
0.884052	0.871121	1.074715	0.9463	0.840867	0.853022

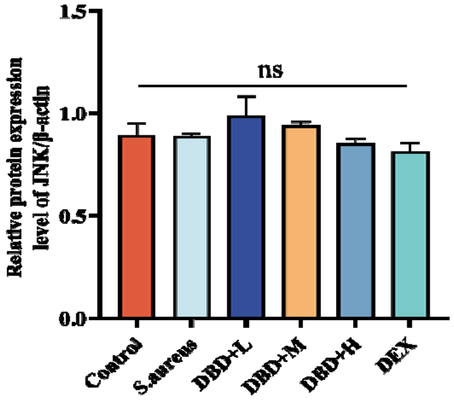

	ERK	p-ERK	
1	26419.62	11645.81	0.440802
2	24757.45	27493.52	1.110515
3	25111.98	25902.93	1.031497
4	26605.4	22764.35	0.855629
5	30931.47	7046.669	0.227816
6	23013.83	22317.59	0.969747
1	26702.76	15222.42	0.570069
2	25763.81	29154.33	1.1316
3	24139.88	26485.52	1.097168
4	25898.81	23429.69	0.904663
5	30445.88	9269.134	0.304446
6	24260	21112.59	0.870263
1	29290.18	15655.18	0.534486
2	22654.86	27648.57	1.220425
3	24401.4	25396.28	1.040771
4	26464.74	24084.98	0.910078
5	31025.23	7922.426	0.255354
6	26395.37	21178.95	0.802374

Control	S.aureus	DBD+L	DBD+M	DBD+H	DEX
0.440802	1.110515	1.031497	0.855629	0.227816	0.969747
0.570069	1.1316	1.097168	0.904663	0.304446	0.870263
0.534486	1.220425	1.040771	0.910078	0.255354	0.802374

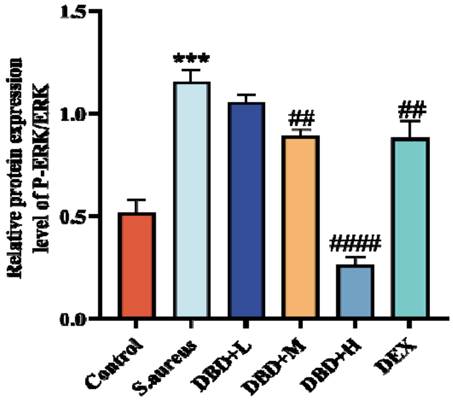

	β-actin	ERK	
1	26014.15	26419.62	1.015586
2	30981.69	29757.45	0.960485
3	23306.15	25111.98	1.077483
4	25177.57	26605.4	1.05671
5	28714.91	30931.47	1.077192
6	25845.18	23013.83	0.89045
1	25388.98	26702.76	1.051746
2	30338.69	27763.81	0.915129
3	22543.15	24139.88	1.07083
4	24946.52	25898.81	1.038173
5	27938.21	30445.88	1.089758
6	25647.47	24260	0.945902
1	27068.93	29290.18	1.082059
2	30887.74	25654.86	0.830584
3	23715.45	24401.4	1.028924
4	25208.1	26464.74	1.04985
5	28129.5	31025.23	1.102943
6	24991.52	26395.37	1.056173

Control	S.aureus	DBD+L	DBD+M	DBD+H	DEX
1.015586	0.960485	1.077483	1.05671	1.077192	0.89045
1.051746	0.915129	1.07083	1.038173	1.089758	0.945902
1.082059	0.830584	1.028924	1.04985	1.102943	1.056173

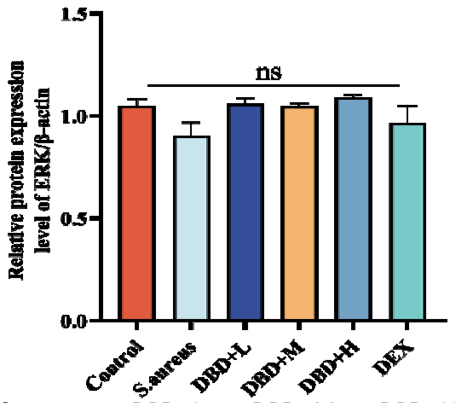

	p38	pp38	
1	25441.05	12589.23	0.494839
2	26324.18	28932.13	1.099071
3	23544	25037.54	1.063436
4	29417.66	22642.64	0.769695
5	17919.64	11027.83	0.615405
6	21994.47	26292.64	1.195421
1	24825.59	13459.71	0.542171
2	26405.18	26870.47	1.017621
3	23091.71	27250.18	1.180085
4	28472.59	20134.1	0.70714
5	17771.64	15683.59	0.882507
6	23303.54	25510.88	1.094721
1	23063.47	9633.154	0.41768
2	24534.05	33531.95	1.366751
3	21277.88	24181.76	1.136474
4	27563	22506.88	0.816561
5	16626.1	10519.57	0.632714
6	21927.13	24349.78	1.110487

Control	S.aureus	DBD+L	DBD+M	DBD+H	DEX
0.494839	1.099071	1.063436	0.769695	0.615405	1.195421
0.542171	1.017621	1.180085	0.70714	0.882507	1.094721
0.41768	1.366751	1.136474	0.816561	0.632714	1.110487

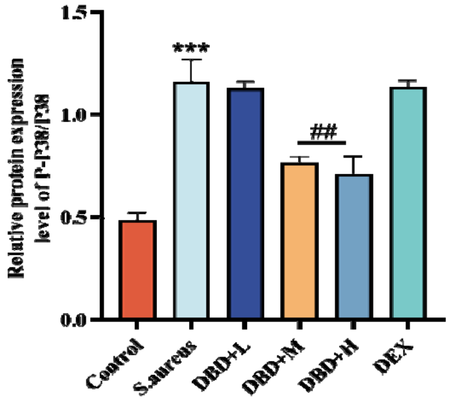

	β-actin	p38	
1	28344.78	25441.05	0.897557
2	30610.13	26324.18	0.859983
3	24175.18	23544	0.973892
4	32984.74	29417.66	0.891857
5	19762.64	17919.64	0.906743
6	21197.81	21994.47	1.037582
1	30533.71	24825.59	0.813055
2	28149.59	26405.18	0.938031
3	27065.05	23091.71	0.853193
4	30580.96	28472.59	0.931056
5	23347.4	17771.64	0.761183
6	25422.18	23303.54	0.916662
1	28852.66	23063.47	0.799353
2	25300.35	24534.05	0.969712
3	25560.1	21277.88	0.832465
4	30915.67	27563	0.891555
5	22490.81	16626.1	0.73924
6	24169.18	21927.13	0.907235

Control	S.aureus	DBD+L	DBD+M	DBD+H	DEX
0.897557	0.859983	0.973892	0.891857	0.906743	1.037582
0.813055	0.938031	0.853193	0.931056	0.761183	0.916662
0.799353	0.969712	0.832465	0.891555	0.73924	0.907235

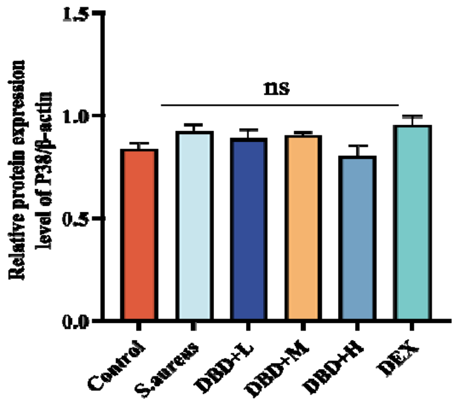

Supplement: Supplementary file 1 [file vetsci-13-00613-s001.zip › WB/WB RAW Data.pdf]
